# Microfluidic Devices: A Tool for Nanoparticle Synthesis and Performance Evaluation

**DOI:** 10.1021/acsnano.3c01117

**Published:** 2023-07-27

**Authors:** Sara Gimondi, Helena Ferreira, Rui L. Reis, Nuno M. Neves

**Affiliations:** † 3B’s Research Group, I3Bs − Research Institute on Biomaterials, Biodegradables and Biomimetics, University of Minho, Headquarters of the European Institute of Excellence on Tissue Engineering and Regenerative Medicine, AvePark, Parque de Ciência e Tecnologia, Zona Industrial da Gandra, 4805-017 Barco, Guimarães, Portugal; ‡ ICVS/3B’s−PT Government Associate Laboratory, 4805-017 Braga, Guimarães, Portugal

**Keywords:** microfluidics, nanomedicine, nanoparticles synthesis, nanoparticles screening, in vitro models, organ-on-a-chip, organisms-on-a-chip, clinical translation

## Abstract

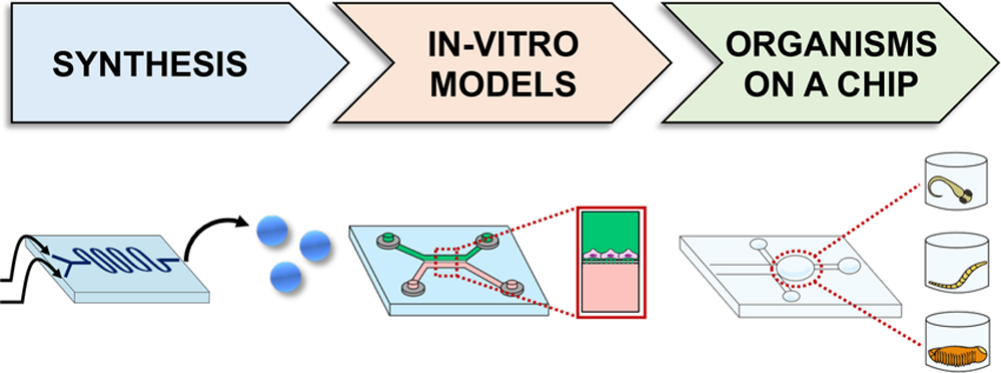

The use of nanoparticles (NPs) in nanomedicine holds great promise for the treatment of diseases for which conventional therapies present serious limitations. Additionally, NPs can drastically improve early diagnosis and follow-up of many disorders. However, to harness their full capabilities, they must be precisely designed, produced, and tested in relevant models. Microfluidic systems can simulate dynamic fluid flows, gradients, specific microenvironments, and multiorgan complexes, providing an efficient and cost-effective approach for both NPs synthesis and screening. Microfluidic technologies allow for the synthesis of NPs under controlled conditions, enhancing batch-to-batch reproducibility. Moreover, due to the versatility of microfluidic devices, it is possible to generate and customize endless platforms for rapid and efficient in vitro and in vivo screening of NPs’ performance. Indeed, microfluidic devices show great potential as advanced systems for small organism manipulation and immobilization. In this review, first we summarize the major microfluidic platforms that allow for controlled NPs synthesis. Next, we will discuss the most innovative microfluidic platforms that enable mimicking in vitro environments as well as give insights into organism-on-a-chip and their promising application for NPs screening. We conclude this review with a critical assessment of the current challenges and possible future directions of microfluidic systems in NPs synthesis and screening to impact the field of nanomedicine.

## 1 Introduction

In the last ten years, developments in the field of nanotechnology led to the production of various types of materials at the nanoscale level. Particularly, nanoparticles (NPs) constitute an exciting mark of this constantly growing innovative field. According to ISO/TS 80,004-1:2015,^1^ NPs are defined as entities with sizes (diameter) ranging between 1 and 100 nm, but in the literature the use of this designation is more frequent for submicrometer particles (1 to 1000 nm). The nanometric dimensions give NPs distinct features. In fact, materials behave differently as their size approaches the atomic scale (atoms and small molecules are around 0.1 and 1 nm, respectively).^2^ This is due to the increased ratio between the surface and the volume (S/V).^3^ Thus, despite nanomaterials’ characteristics (e.g., size, surface potential, etc.) being strictly related to the bulk material used for their production,^4−6^ the physical, chemical, and biological properties of a material engineered at the nanometric or larger scale will differ.

NPs are widely used in nanomedicine due to their potential to impact several medical fields. They can be used for early detection, diagnosis, treatment, and follow-up of different diseases. NPs can be generated from several bulk materials (both organic and inorganic) and are very attractive, as they are extremely versatile devices. For diagnosis, these engineered nanomaterials can contain different probes for imaging purposes or interact with specific biomolecules (e.g., cancer biomarkers).^7^ In therapeutics, NPs increase a drug’s bioavailability and target specificity, reducing its side effects (e.g., systemic and organ toxicity).^8,9^ In fact, NPs’ shape, size, and surface can be tailored to achieve passive and active targeted-drug delivery. Additionally, they can have stimuli-responsive properties (e.g., pH, temperature, hypoxia, or redox potential) to allow drug release only if a specific pathological or biological trigger is present.^10,11^ Despite the countless advantages that NPs offer, only a very small number of them were approved by the U.S. Food and Drug Administration (FDA) and/or European Medicines Agency (EMA).^12^ The majority of the approved formulations are phospholipid-based carriers (liposomes), followed by polymeric NPs, which are mainly used in cancer treatment. There are also in the clinic inorganic NPs, predominantly, iron-based NPs for both therapy and imaging.^13−15^ As a result of the success of these nanoformulations, considerable efforts continue to be made to increase their number in the clinic through a large number of ongoing clinical trials.^16^ As a consequence, the demand for NPs with outstanding properties and in vitro models that provide better extrapolation to the human scenario has grown extensively.

A hallmark of NPs’ performance is their physicochemical properties that are closely related to the methods used in their production. Hence, this development step represents one of the greatest challenges in this field. In this context, microfluidics has acquired huge importance over the last years, as a branch of science and technology that allows accurate manipulation and monitoring of the fluids on micrometric scale channels.^17−19^ Microfluidic devices have applications in several areas, including chemical synthesis,^20^ molecular biology,^21^ tissue engineering,^22^ and NP screening in terms of transport and efficiency.^23^ Due to countless advances and innovations, microfluidic devices are expected to be the key to improving the controlled synthesis of NPs and accelerating their transition to clinical evaluation. The employment of these tools for NP production provides several advantages compared to conventional batch synthesis such as (i) to foresee identical reaction conditions along the production method, ensuring high reproducibility;^24^ (ii) improved cost efficiency and ecofriendly impact due to the use of low amounts of environmentally friendly solvents;^25^ (iii) high level of control over experimental parameters that lead to NP size uniformity;^26^ (iv) enhanced mixing within the channels;^27^ (v) reduced synthesis time;^28^ (vi) possible automation of the system that results in a reduction of manual errors;^29^ and (vii) endless geometries can be produced and customized based on specific needs.^30^

**Figure 1:**
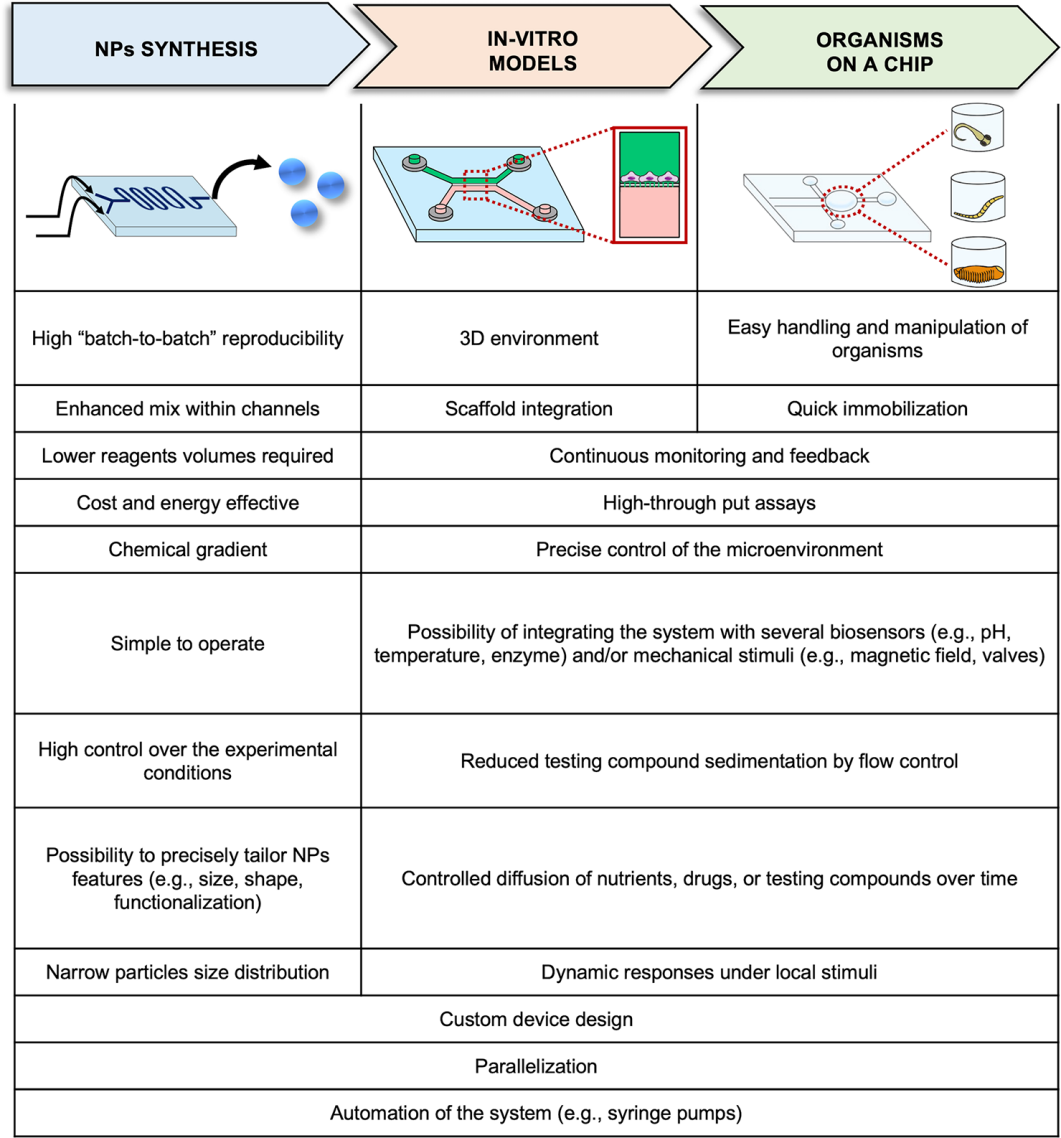
Scheme illustrating the microfluidics application in NPs synthesis, in vitro models, and organism-on-a-chip and their advantages. To date, microfluidics technologies allowed improvement of the NP synthesis process and in vitro and in vivo screening through the manipulation of, respectively, 3D cell cultures and small organisms, such as *Caenorhabditis elegans* worms, *Drosophila melanogaster*, and *Danio rerio* larvae inside microfluidic devices.

As mentioned above, over the years, microfluidic devices have proved to be a powerful tool not only to produce NPs but also for their testing (Figure 1). The extensive research and growth of the microfluidic field have been driven by the ability of these devices to process small volumes of samples (micro- to picoliters), being able to mimic a biologically relevant length scale. Indeed, microfluidics is also successfully employed in in vitro assays.^31^ These devices present channels with specific geometries to mimic different environments and inlets and outlets for cell seeding, culture, sampling, and analysis.^32^ These microphysiological devices are intended to mirror the functions of a specific tissue, organ, or physiopathological condition to serve as a model for in vitro studies.^33,34^ Furthermore, the addition of three-dimensional (3D) structures (e.g., hydrogels and scaffolds) or cell aggregates (e.g., spheroids or organoids) allows for obtaining more elaborate models.^35,36^ The 3D culture of cells and the application of a dynamic environment (e.g., perfusion, shear stress) better represent tissues’ nature and lead to more reliable outcomes than conventional two-dimensional (2D) static cell cultures.^37^ Finally, microfluidic chips can also be built to host small organisms such as *Caenorhabditis elegans* worms, *Drosophila melanogaster,* and larvae of *Danio rerio*.^38−40^ Microfluidic chips also allow for the manipulation of these small animals with care and precision, eliminating their potential damage due to mishandling.^41^ These in vivo models provide great opportunities for drug screening as well as efficacy and toxicity evaluation. Moreover, the integration of small organisms on a chip guarantees high control over the experimental conditions and enables data processing in parallel, generating high-throughput data.

Despite the growing interest in microfluidics to advance the nanotechnology field, a comprehensive literature review covering the synthesis, testing, and application of NPs using microfluidic devices is lacking. Previous reviews focus mainly on specific aspects, such as microfluidic devices for NPs synthesis and/or organs-on-a-chip.^42−44^ They fail to establish the link between them and do not explore the realm of organisms-on-a-chip. Accordingly, our review starts with an exhaustive and up-to-date evaluation of the use of microfluidic technology in NP synthesis. Next, it delves into the potential of microfluidic devices to replicate physiological conditions and their advantages for in vitro testing of NPs. Lastly, it addresses a relatively unexplored area, organisms-on-a-chip, specifically focusing on its relevance to advance safety and efficacy evaluation. Finally, current challenges and future research directions for this quickly evolving field are presented.

## 2 Microfluidic Devices

Microfluidic devices can be strategically designed and produced to meet different flow patterns and, therefore, applications. Microfluidic devices were initially made of silicon or glass and were manufactured using micromachining techniques.^45^ This area of mechanical engineering involves the use of different techniques (e.g., wet/dry etching, photolithography, electron beam lithography, etc.) that allow building microstructures by engraving the desired pattern into the material.^46^ However, these techniques require the use of clean-room facilities and expensive production equipment that translate into high costs. With the introduction of materials such as polymers, the prevailing method for manufacturing a microfluidic device is soft lithography. Other techniques were also investigated to improve the fabrication of the microreactors, such as microcutting,^47^ photolithography,^48^ laser ablation,^49^ 3D printing,^50^ plasma etching,^51^ injection molding,^52^ and hot embossing.^53^ Each of these techniques offers advantages and may be suitable for specific applications or materials. For instance, 3D printing allows for the rapid prototyping of complex microfluidic structures with high precision.^54^ For a more comprehensive understanding of the cutting-edge technologies employed in the fabrication of microfluidic devices, there is recent literature that provides detailed analysis and insights into these advancements.^55−60^

The manufacturing technique used to produce microreactors is strictly related to the materials used in their fabrication. As mentioned before, silicon and glass were among the initial materials utilized for the production of microfluidic devices. Glass is optically transparent and electrically insulating, while silicon is opaque and a semiconductor. Moreover, they present high resistance to organic solvents, high thermal conductivity, and stable electroosmotic mobility. However, they have some limitations, such as the need to use hazardous substances during the manufacturing process, and their hardness and brittleness make the bonding step challenging.^61^ Finally, both materials are impermeable to gases, being, for instance, not suitable for cell culture applications. Other inorganic materials, such as quartz and ceramic, can be used to produce microfluidics, but they present similar limitations. Additionally, they are costly, and their handling usually requires skilled technicians and expensive facilities due to the dangerous chemicals involved in their processing. Advantageously, technological advances occurred over the years, and advanced materials, including polymer substrates, paper, or composites, were used for microfluidic chip production. Polymeric materials were introduced due to their great flexibility and low cost in the production of microfluidics devices.^62^ Elastomers are the most employed polymers in this area. Some examples are polydimethylsiloxane (PDMS), thermoset polyester (TPE), and thermoplastic polymers (e.g., polystyrene, polycarbonate, poly(methyl methacrylate), polyethylene glycol diacrylate, and polyurethane). These polymers generally have good optical transparency, elasticity, and gas permeability, but their application is limited due to the aging of the material, poor resistance to high pressure, and chemical compatibility with many organic solvents.^63^ Therefore, they are mainly used for the manufacture of cell culture devices for in vitro models.

Paper is another flexible organic compound that was recently explored.^64^ This cellulose-based material has great potential due to its flexibility and biocompatibility. Moreover, it can be modified by the incorporation of nitrocellulose or through surface chemistry modification. Indeed, by applying water-insoluble oxidants, it is possible to produce a microfluidic paper-based analytical device for the assessment of reducing substances.^65^ Paper-based microfluidics relies on a passive mechanism that pulls the solutions through the device by capillarity. This system can also be conjugated with polymers, creating a paper/polymer hybrid microfluidic chip that is mainly employed for enzyme-linked immunosorbent assays (ELISA).^66^ However, the applications of paper-based microfluidic devices are limited compared with traditional microfluidic devices. For instance, paper devices, being not optically transparent, are not suitable for absorbance spectroscopy. Moreover, paper channels are not compatible with the cell culture and droplet generation. Finally, the layout of the paper fibers can vary dramatically, and sample recovery is impractical because it is absorbed into fibers.^67^

In summary, the material and geometry of the microfluidic device dictate the chip properties. As such, it is extremely important to take into account the end application when selecting or producing a microreactor.

### 2.1 Microfluidic Devices for NPs Synthesis

**Figure 2:**
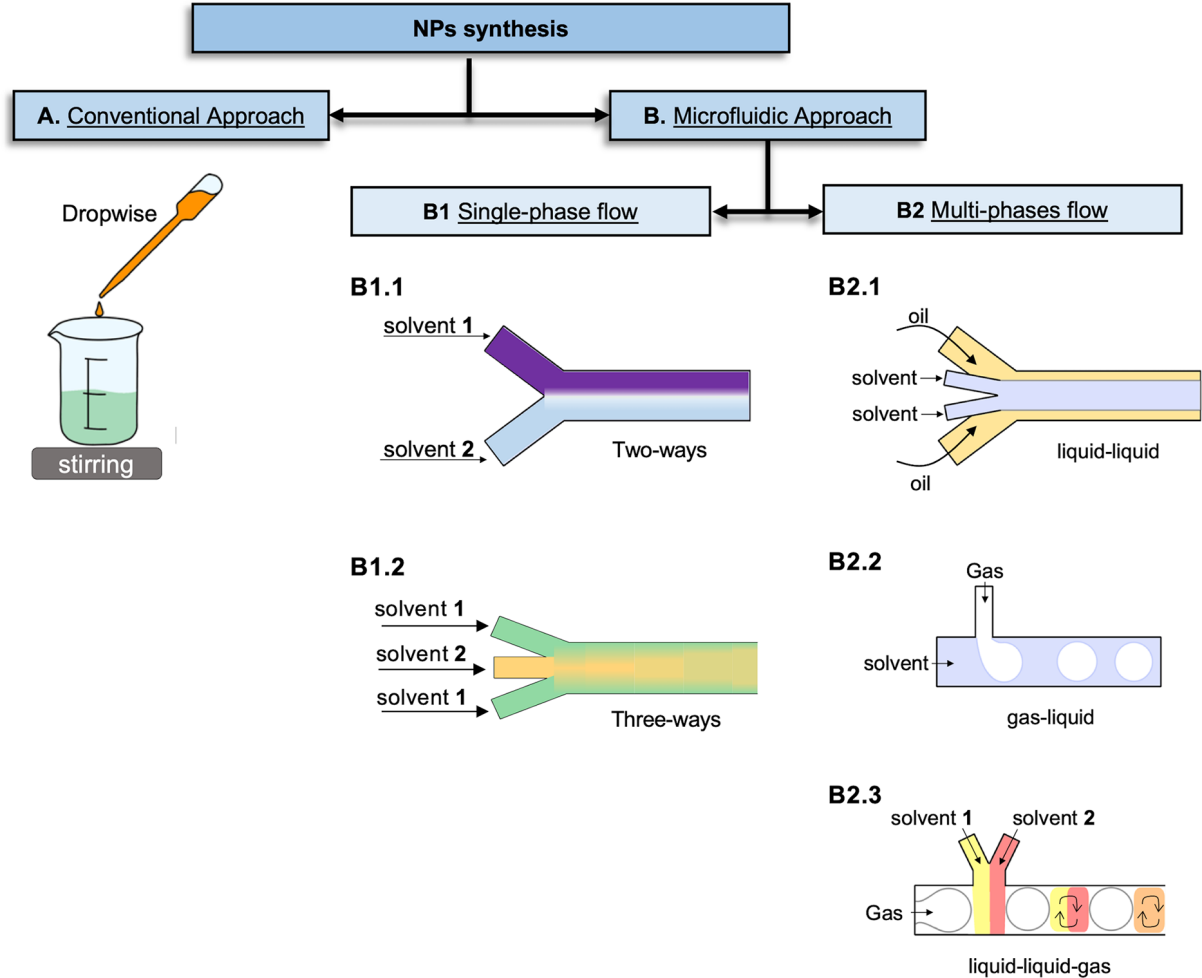
Schematic representation of one of the most used conventional approaches for NP generation, the dropwise method (A). Microfluidic chips (B) with different designs can be employed for NP production based on the type of flow used, namely, single-phase flow (B1) with two- (B1.1) or three-way channels (B1.2), and multiphase flow systems (B2), such as the liquid–liquid (B2.1), the gas–liquid (B2.2), and the liquid–liquid-gas (B2.3).

Microfluidics has rapidly evolved as one of the most promising platforms for NPs synthesis. Indeed, they allow for generating products with superior performance and properties compared to the conventional methods, such as the dropwise method, one of the most used approaches for NPs generation (Figure 2A). Hence, microfluidic systems (Figure 2B) were demonstrated to be highly suitable for the controlled synthesis of NPs.^68^ The precise control of NPs physicochemical properties is crucial to obtain the desired therapeutic effects. Conversely, for the most common and traditional synthesis processes, microfluidics allows for precisely controlling the resulting NPs properties. Thus, it enables the creation of NPs tailored to specific applications, such as drug delivery, by achieving formulations with the desired characteristics. For instance, the ability to finely tune experimental parameters allows for the production of highly monodisperse NPs, ensuring consistent properties within or between different batches. The importance of uniformity becomes particularly significant in applications where particle characteristics directly influence performance or desired outcomes as in nanomedicine.^69^ Furthermore, microfluidics advances have facilitated in situ NPs characterization by seamlessly integrating advanced analysis techniques, such as synchrotron small-angle X-ray scattering (SAXS).^70^ This emerging application involves the combination of specially designed microfluidic devices with SAXS, creating a platform that allows for real-time detection of dynamic structural changes during the production of NPs.^71,72^ This approach offers a more comprehensive understanding of the nucleation and growth mechanisms involved in the formation of NPs within confined geometries.^73^ Overall, miniaturization of the NPs synthesis process offers several advantages, as presented in Figure 1. For instance, microfluidic devices allow improving mixing and speeding up chemical reactions that take place in the micrometric channels, which leads to the generation of homogeneous NPs. Moreover, the experimental parameters can be easily controlled to generate NPs with defined features, such as (i) the volumetric flow rate, which usually is represented by the symbol Q and is defined by the volume of fluid that passes through a channel per unit time; (ii) the total flow rate (TFR), which is the sum of flow rates entering a microchannel; (iii) the flow rate ratio (FRR), which is the ratio between the flow rates of the organic and aqueous solutions inside the channel, and it is a dimensionless value; (iv) the concentration of reagents; (v) the pH; and (vi) the temperature. In particular, the parameters governing the flow rate are closely linked to polymer concentration and NP residence time in microfluidic devices. Additionally, FRR has a high impact on the NPs’ size and polydispersity index (PDI). As the FRR increases, the width of the organic stream carrying the NPs precursor decreases, resulting in enhanced diffusion between the streams. As a consequence, the mixing time decreases, ultimately leading to the production of smaller NPs.^68^ Moreover, pH has demonstrated the ability to impact NP synthesis and can be employed to tune the resulting size.^74^ For instance, the use of a more acidic buffer led to the production of smaller liposomes.^75^

The temperature can also accelerate chemical reactions, improve fluid mixing, and, thus, influence NPs’ size. For instance, silver NPs increased in size when microdroplets were subjected to an increased heating time (60 °C for 0, 1, 2, 4, 6, and 8 h).^76^ Conversely, smaller lipid NPs were obtained when the temperature was set at 47 °C compared to those synthesized at 21 °C.^77^

Despite accepting that the use of microfluidic systems for NP synthesis offers numerous advantages, it is equally important to acknowledge their limitations. For example, handling and using devices with such small dimensions can be challenging. The micrometric scale of the channels can make their cleaning difficult and often leads to clogging, particularly if intricate geometries are present. Other limitations are the cost that these devices can present and the specialized additional equipment that may be required, such as temperature sensors,^78^ magnetic fields,^79^ ultrasound systems,^80^ alternating current,^81^ and automated syringe pumps.^82^ Additionally, the scalability of the NP synthesis process presents a challenge, although recent efforts have been made to address this issue, as discussed in Section 2.1.3.

In microfluidics, the flow of a fluid across the microsized channels can be calculated by the Reynolds number (Re), which is described by the ratio of inertial forces to viscous forces, as the following equation:^83^
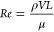
where ρ is the density (kg/m^3^), *V* is the drift velocity (m/s), *L* is the diameter of the inlet channel (m), and μ is the dynamic viscosity of the solvents [kg/(m·s)].

Based on the API 13D recommendations,^84^ the Re can be used to classify the fluid systems into three categories, namely, (i) laminar flow (Re < 2000); (ii) critical flow (2000 < Re < 4000); and (iii) turbulent flow (Re > 4000). The laminar flow is characterized by a smooth and regular path of the fluids. The critical flow can be used to define the transition from laminar to turbulent fluids, which is defined by irregular fluctuations in the pressure and flow velocity of the liquid. However, due to the microsized dimensions of the channels, the Re value in a microfluidic device is usually less than 100. As a consequence, these devices exhibit laminar flow of fluids, which leads to improved heat and mass transfer capacities.^85^

Various microfluidic devices can find applications in the synthesis of NPs. For instance, to produce PLGA–PEG NPs, flow-focusing devices,^86^ micromixer,^68^ or multiphase systems^87^ can be employed. Thus, it is crucial to explore multiple options and evaluate the suitability of the device based on the desired NPs features and research goal. In the following sections, we will explore the two primary categories of microfluidic devices: single-phase (Figure 2B1) and multiphase flow systems (Figure 2B2), presenting examples of their applications.

#### 2.1.1 Single-Phase Flow Systems

The single-phase flow systems (Figure 2B1) are the most commonly used for NP generation by nanoprecipitation and self-assembly processes. Indeed, they proved over the years their ability to enhance the controllability, reproducibility, and homogeneity of the environment during the reaction that aids the generation of NPs with a narrow size distribution.^88^

**Figure 3:**
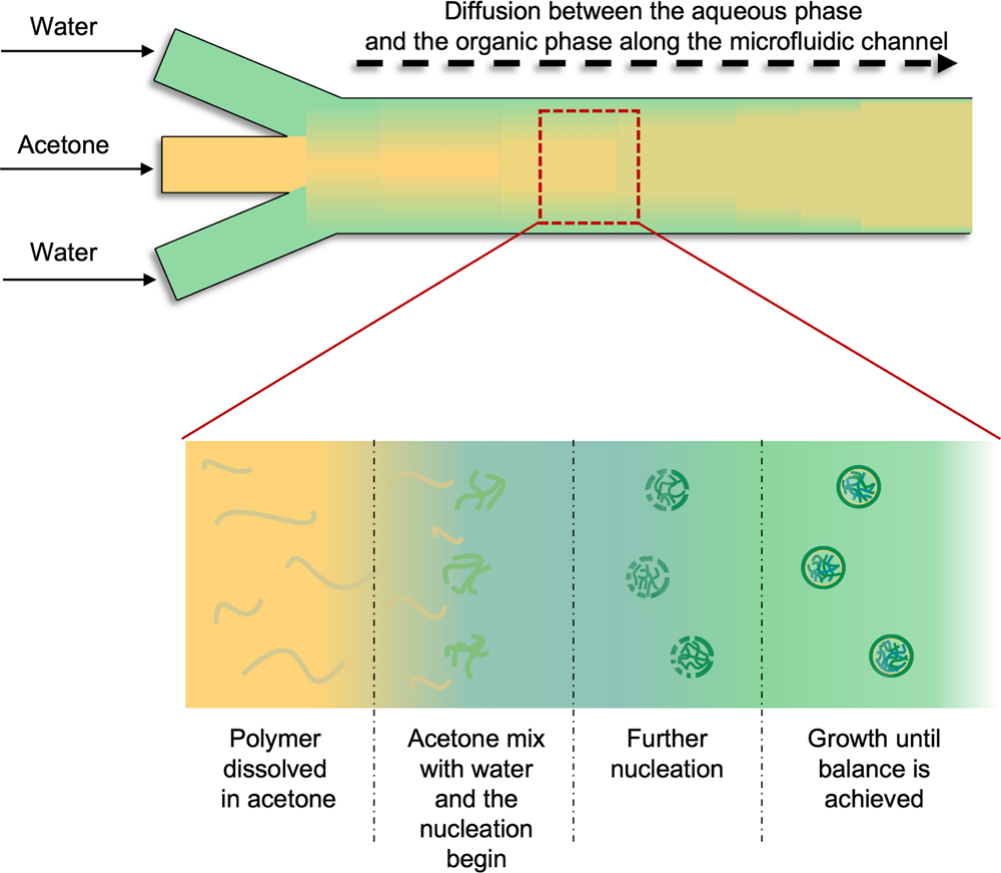
Mixing process inside a linear microfluidic chip between two miscible solvents, such as water and acetone, occurs due to the diffusion of the acetone into the water, generating a homogeneous solution along the channel. For nanoprecipitation, a hydrophobic polymer soluble in an organic solvent (acetone) can precipitate due to its poor solubility in water. Consequently, as the diffusion progresses, the polymer chains collapse on themselves and aggregate (nucleation phase) into NPs. The rapid mix improves the reaction of nucleation and growth, until the balance is achieved and uniform NPs are generated.

The NP synthesis in these systems consists of the establishment of a laminar flow between single or multiple miscible fluid streams through the device channels, where nucleation and growth occur (Figure 3). Indeed, in the nanoprecipitation process, NP formation occurs through diffusion-nucleation-growth. In these devices, the mixing happens by diffusion across laminar flow streams, as no turbulent regimes are generated. The laminar flow occurs when the mixed liquids flow smoothly in parallel layers. When this condition is established, the fluid flow is steady, and it is characterized by high lateral diffusion and rare episodes of convections.^89^

For example, a single microfluidic device was recently used for synthesis of pH-responsive polymeric micelles by coflow nanoprecipitation.^90^ The geometry of the employed device allows the organic phase to flow into the internal channel, while the aqueous solution flows in the external capillaries in the same direction. The mixing in the microfluidic device is enhanced, and the time needed for nucleation and growth is reduced. The obtained micelles presented dimensions below 170 nm with a narrow distribution range. In another study,^91^ an X-junction with three inlets and a single outlet channel was used to produce PEGylated-hyaluronic acid NPs, exploiting the hydrodynamic flow-focusing approach. Conversely to the previous example, in this system, the aqueous solution flows in the middle channel, while the organic solution is injected in the side channels. The feasibility of the study was carried out by exploring different parameters, such as FRR, temperature, and molar ratio between reactional functional groups of the cross-linking reaction. The system proved its value in the production of NPs ranging from 30 to 800 nm with higher stability in water compared to the conventional batch mode. Another hydrodynamic flow-focusing microfluidic was used to produce gelatin NPs and evaluate their in vitro performance.^92^ This approach allows a drastic reduction in the size of NPs compared to the bulk preparation methods, generating particles of ∼10 nm. A single-phase microfluidic device was also employed for the synthesis of gold (Au) nanorods.^93^ The presented system allowed controlling of the seed formation and nanorod growth. Moreover, it increased reproducibility and allowed on-stream polymeric coating with a 100-fold reduction of the reagent consumption compared to the conventional batch approach. Also, this device aided the precise tuning of the ionic modifier concentrations (Cl^–^ and Br^–^) that allow tailoring the shape of the resulting rods.

The employment of single-phase flow systems in nanoprecipitation and self-assembly processes for NP generation has proven to be highly effective. These systems have the capacity to enhance mixing, reduce nucleation and growth times, and significantly improve control, reproducibility, and homogeneity of the resulting NPs’ properties. Thus, NPs with precise dimensions have been produced. The establishment of laminar flow within these systems allows for efficient mixing by enabling diffusion across parallel layers, thereby eliminating the occurrence of turbulent regimes. The main limitation in using linear single-phase flow reactors is the slow diffusion that occurs in laminar flow, which restricts the reaction speed. Moreover, it is characterized by a parabolic velocity flow profile that causes an uneven distribution of the residence time along the channel that might translate into an increased NP size distribution. To overcome these shortcomings, a valid option is to employ micromixer devices that help to improve the single-phase flow reactors’ performances, for instance, by forming disturbance inflow via folding and bending to enhance the mix.

**Figure 4:**
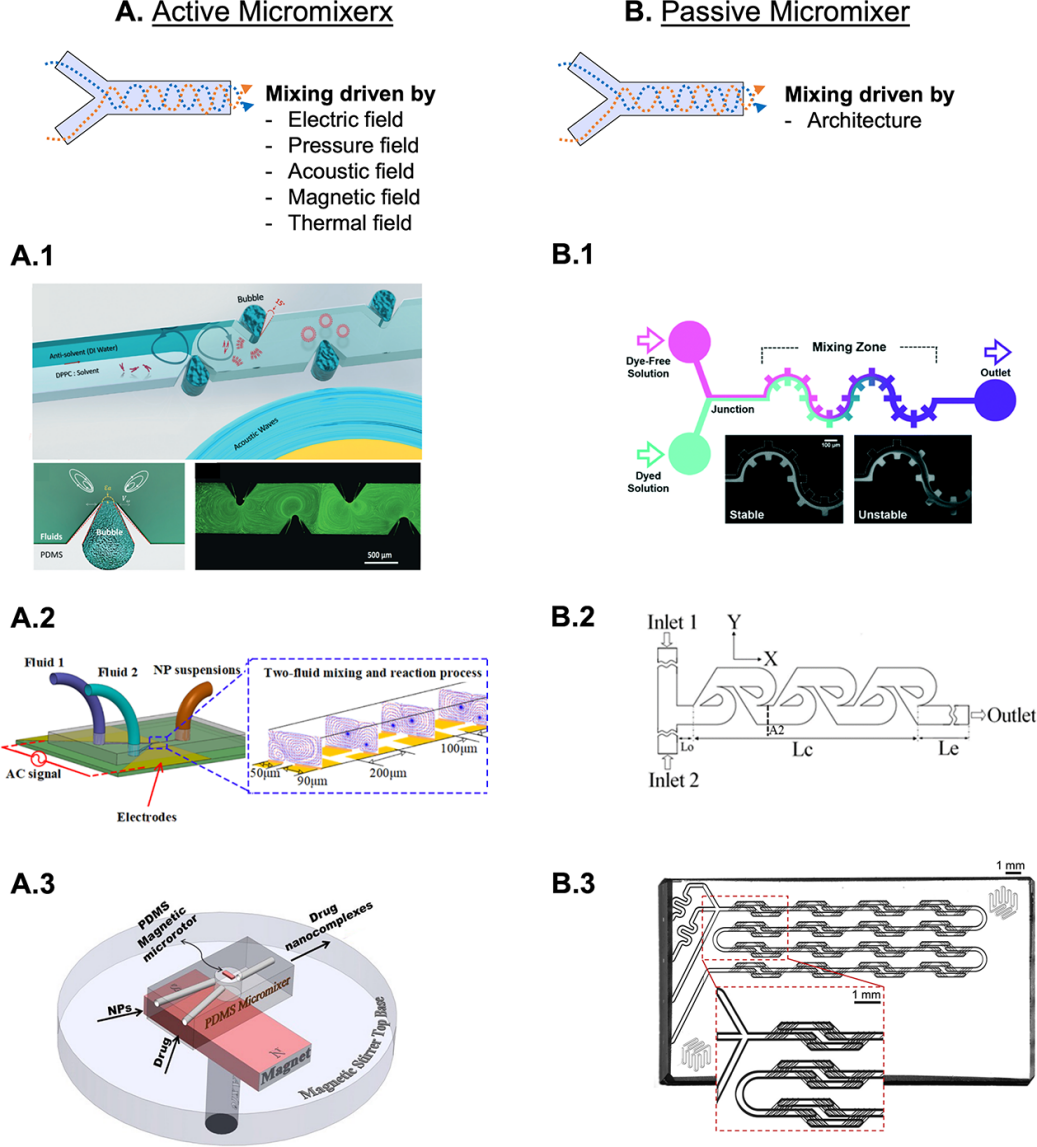
Examples of active micromixers (A) where the mixing of the injected fluids is induced by acoustic waves (A.1 - Adapted with permission from ref (94). Copyright 2019 Royal Society of Chemistry), alternating current electrothermal field (A.2 - Adapted with permission from ref (96). Copyright 2020 American Chemical Society), or magnetic field (A.3 - Adapted with permission from ref (98). Copyright 2016 Elsevier). In the passive devices (B), the mix is achieved due to the architecture of the channels, such as the gear shape (B.1 - Reprinted with permission from ref (99). Copyright 2021 Royal Society of Chemistry), the tesla (B.2 - Reprinted with permission from ref (100). Copyright 2010 Elsevier), and the herringbone (B.3) micromixers.

Micromixers can be divided into active or passive microfluidic devices (Figure 4A,B). Active devices employ external energy sources, such as electric, pressure, acoustic, magnetic, or thermal fields, to enhance the quality of the mixing. For instance, in order to achieve homogeneous nucleation, an acoustic-driven micromixer that integrates sharp edges and bubbles in its channel design was explored to magnify the amplitude of vibration, enhancing the mixing speed and homogeneity of the resulting polymeric NPs (Figure 4A.1).^94^ This work is a proof of concept regarding the application of acoustic-assisted micromixers in NPs synthesis. By altering the mixing time, the nucleation process was manipulated to tune the NPs size. Results showed how the use of this micromixer device resulted in smaller NPs compared with the ones obtained by passive hydrodynamic flow focusing. Acoustic-driven micromixers also demonstrated high throughput performances.^95^ Indeed, the proposed device consists of a micromechanical oscillator, placed between two channels that guide the fluids. Once the optimal frequency is achieved, this system can mix two fluids within 4.1 ms with an efficiency of ∼91%. This platform proved its versatility, guiding the synthesis of budesonide NPs and DNA NPs with an average diameter of ∼63 and 80 nm, respectively.

Additionally, a sequential micromixing-assisted process was achieved by the application of an alternating current electrothermal field to produce inorganic NPs (Figure 4 A.2).^96^ This peculiar electric field exerts a force on the fluids, inducing vortex motions. It was demonstrated to induce efficient mixing between fluids, resulting in NPs with a narrower size distribution and smaller average size (100 nm average cubic NPs) in comparison with the traditional mechanical mixing. Another kind of electric field was explored to produce liposomes. Indeed, electrohydrodynamic-driven micromixing was explored in this context.^97^ In this study, the proposed active micromixer features microelectrodes that induce an electric field transverse to the solvent (ethanol) and antisolvent (water) streams. When low alternating current voltages are applied, discontinuities are created at the interface between the two streams which drive the movement of the fluids and determine an efficient mixing and consequent nanoprecipitation, which leads to the formation of highly monodisperse liposomes. Interestingly, the mechanism of this active micromixer makes it a very versatile tool that can be employed to produce different NPs based on the mixing of biphasic liquids.

Magnetically active micromixer may assist the synthesis of drug nanocomplexes^98^ (Figure 4A.3). For that, a magnetic microrotor generated rotations in the chamber of a micromixer and induced vortice motion, which aids the maximum load of a drug (benzathine penicillin G tetrahydrate) in titanium dioxide (TiO_2_) NPs. The NPs were found to be hydrophilic and negatively charged with ∼38 wt % drug conjugation, which effectively annihilated the bacteria similar to the treatment with 100 wt % of the free drug. Overall, the examples presented above showcase the significant potential of active micromixers in enhancing NPs synthesis. These devices have demonstrated the ability to improve mixing efficiency, resulting in the production of NPs with enhanced features when compared to alternative methods. Although it is important to consider the associated costs and feasibility of implementing external energy sources.

Conversely to active micromixers, passive devices do not rely on any external actuator to drive the fluid streams. Indeed, their mixing is mainly increased by an enhancement of the contact surface between the fluids. To achieve this goal, the key feature is the geometry of the channels. Many special architectures were explored over the years for passive micromixers. Some examples include parallel and multilaminations, obstacle-channel, curved-channel, serpentine, herringbone, and unsymmetrical geometries. For instance, a gear-shaped micromixer was used for the synthesis of silica NPs (Figure 4B.1).^99^ In this work, the authors proposed a passive micromixing technique utilizing the inertia-elastic flow instability that takes place in a low-viscosity polymer solution in a serpentine design channel. This design significantly enhanced the mixing in the gear-shaped channel, leading to more homogeneous silica NPs populations with reduced energy consumption. Additionally, a tesla-micromixer was used to produce antigen-coated NPs (Figure 4B.2).^101^ This peculiar structure effectively enhanced the mixing of fluids by inducing transversal convection, leading to NPs with smaller sizes, higher monodispersity, and reproducibility. Tesla’s micromixer efficiency usually relies on the asymmetric structure of the channel or the flow rate ratio of the fluids. Thus, tesla’s micromixer with several geometries has been explored to enhance the mixing efficiency and chemical reactions.^102^ Another efficient architecture is represented by the herringbone-like device used by our group to develop both polymeric and polysaccharide-based NPs (Figure 4B.3). This micromixer generates laminar streams, which allow for improving the surface area between the organic and aqueous phases by generating several layers of fluids. Due to its geometry, the streams are forced to split and rearrange together at each mixing stage inside the device. This continuous stretching, folding, splitting, and recombination of the fluids dramatically reduce the diffusion distances, which translate to an improved mixing time and leads to the production of NPs with relatively high monodispersity level and fine-tuning of their sizes.^68^ Additionally, we demonstrated the versatility of this device by generating polysaccharide complexes with a size of around 100 nm compared to the dropwise method that generated ∼2 times bigger NPs. Our study suggests that the synthesis method affects the polysaccharides’ arrangement during NP complexation and, in particular, their sizes.^103^

Passive micromixers have valuable applications in the synthesis of hybrid NPs that incorporate multiple materials to attain distinct properties or functionalities. Particularly, lipid-polymer hybrid NPs emerged as advanced drug delivery systems.^104,105^ Also in this field, microfluidics show potential. For instance, by leveraging a specifically designed microfluidic device, hybrid NPs were successfully fabricated, featuring polymer cores and lipid-monolayer or lipid-bilayer shells. This innovative approach facilitated the production of hybrid NPs with varying flexibility and energy dissipation, which can lead to distinct interactions with cells.^106^

In recent years, passive micromixer devices have emerged as valuable tools for synthesizing lipid NPs to encapsulate nucleic acids, particularly in the context of vaccine application. For instance, the iLiNP device was specifically designed for the synthesis of lipid NPs loading nucleic acids.^107−109^ Fabricated using photolithography, this device incorporates 3D grooved mixer structures. The inclusion of baffle structures within the device significantly enhanced solution mixing, allowing for precise size tuning at intervals of 10 nm within a diameter range of 20 to 100 nm. This achievement marks a significant advancement, as such precise size control had not been achieved previously. Furthermore, the efficacy of the iLiNP device in producing lipid NPs loaded with siRNA was evaluated through in vivo experiments. The developed lipid NPs demonstrated the ability to effectively deliver siRNA to hepatocytes and exhibited notable therapeutic activity. This highlights the potential of the iLiNP device as a valuable tool for siRNA-loaded lipid NPs production.

#### 2.1.2 Multiphase Flow Systems

Microfluidic devices with multiphase flows work on segmented streams of two or more immiscible phases. This phenomenon, characterized by the alternation of successive segments, is called a segmented flow. The flow phases can be liquid–liquid, liquid–liquid-gas, or gas–liquid flows (Figure 2 B2). The interaction between immiscible phases combined with the applied forces results in a flow characterized by peculiar streams that can be divided into bubbly, slug or Taylor, churn, annular and slug-annular profiles.^110^ The most used flow pattern is the segmented flow, also known as Taylor flow, which is characterized by droplets surrounded by liquid. Droplet-based microfluidic platforms were used to produce various NPs including Au nanostars,^111^ lead sulfide quantum dots,^112^ and metal nanocrystals.^113^ Additionally, these devices were successfully used in synthesizing NPs with asymmetry or a heterogeneous nature, such as Janus NPs (JNPs). Indeed, the precise control of the droplet volume and the reliable manipulation of individual droplets during synthesis enabled the production of anisotropic Au-nanorod@Ag-polyaniline JNPs with uniform size and excellent dispersion.^114^ Indeed, the droplet-based microfluidic platform allowed for enhancing reproducibility, automation, and precise control over the synthesis process compared with the most established bulk methods.

The flow segments generated within the channel act as reaction chambers, where mixing occurs as the segments move along the channels, reducing the risk of clogging and enhancing molecular interactions. In fact, the variability of these systems allows for enhanced mixing and mass transfer, while it reduces the residence time and the reagents deposition on the channel walls.^115^ Due to the characteristic microscale dimension of these systems, some physical parameters (e.g., shear viscosity, coefficient of diffusion, and surface tension) acquire a more powerful impact and may prevail over the gravitational and inertial forces that are dominant in macroscopic flows. This characteristic sets multiphase flow devices apart, as they are extensively utilized for NPs synthesis. Moreover, the configuration and arrangement of microchannels in specific patterns allow for optimal mixing and reaction time, making geometry a key player in the device’s efficiency. For instance, a droplet-based microreactor was employed to study the effect of different flow rates on the properties of magnetic NPs.^116^ The device presented different patterns that work as multifunctional units (T-junction, Y-junction, and S-channels). This multiphase flow device worked by generating droplets containing different reagents that are subsequently fused and mixed by stretching and folding through the S-shaped region to enhance the mixing reaction. This approach allowed the synthesis of magnetic NPs with a high control over the oil and aqueous flow rates. The NPs obtained by coprecipitation showed superparamagnetic behavior and a size increase from 17 ± 5 nm to 29 ± 4 nm.

In gas–liquid microreactors, carbon monoxide (CO) and carbon dioxide (CO_2_) are the most commonly employed gases. For instance, Au NPs were produced in a coiled flow inverter (CFI) reactor, using CO as the reducing agent.^117^ Several capping agents [trisodium citrate, polysorbate 80, oleylamine, and poly(ethylene glycol) 2-mercaptoethyl ether acetic acid] and operational parameters were evaluated on the resulting NPs size and PDI. This gas–liquid reactor was constituted of 100 coils. Each section contained 5 coils and presented a curvature of 90°, forming a compact design that allowed for the generation of highly monodispersed NPs. The segmented flow was generated by the insufflation of CO in the stream of an aqueous solution of a chloroauric acid (HAuCl_4_) through a T-junction. Due to the hydrophobicity of the wall, isolated liquid snails were generated inside the channel, resulting in a plug flow. This system led to the synthesis of monodisperse Au NPs with sizes lower than 10 nm, showing the possibility of fine-tuning the NPs’ size and hydrophilicity by capping agents. Additionally, a three-phase reactor that allows for the repeated and controlled addition of reagents into droplets was produced.^118^ The system used for the synthesis of quantum dots consisted of an alternating steam of argon gas and octadecene droplets dispersed in an immiscible liquid carrier (perfluorinated polyether). The argon gas injection maintains uniform spacing between droplets, while the T-junctions, present along the channel, can be used to repeatedly inject another reagent inside the previously generated droplets. This led to precise control over the growth reaction by allowing for multiple additions of the reagents. Indeed, the reported results demonstrated differences in the resulting particle volume means (around 23 nm^3^ vs 43 nm^3^) in the case of single or multiple-addition experiments. Finally, a liquid–liquid-gas multiphase flow device was developed for continuous plasmonic NPs synthesis.^119^ The microfluidic device was built to induce a segmented flow at the microfluidic T-junction. It contained alternately aqueous solutions for the synthesis of NPs, and gas bubbles were dispersed in an immiscible oil phase flowing within the microchannel. Gas was injected periodically to stop further entry of reagents, thus preventing the buildup and deposition that can occur when the laminar flow is constant. This innovative feature prevents undesirable events, such as droplet coalescence, which commonly occur in current droplet-based synthesis methods. This simple platform allowed for a robust mixing with no operator intervention, generating monodisperse NPs with no postsynthesis treatment required.

Ultimately, passive micromixers have been shown to be a promising alternative to active micromixers by leveraging on the enhanced quality of contact surfaces between fluids without the requirement of external actuators. Several geometric shapes have been investigated with the goal of improving mixing efficiency and producing NPs with desirable properties.

#### 2.1.3 Microfluidic Devices to Scale up NPs Synthesis

In recent years, there has been growing interest in exploring microfluidic technologies to scale up the production of NPs. The aim is to harness the precise synthesis capabilities offered by microfluidics on a large scale and make them accessible to, for instance, the pharmaceutical industry. However, the large-scale production of NPs by using microfluidic technology faces several key limitations. One of these challenges is the limited scalability of the microfluidic setup. Indeed, microfluidic systems are typically characterized by low flow rates and small reaction volumes, resulting in a limited production throughput. Moreover, the complex fluid dynamics and control systems involved in microfluidic devices can be difficult to replicate and maintain while working with large volumes. Another challenge is linked to process optimization. Microfluidic platforms often require meticulous optimization of various parameters (e.g., flow rates, flow rate ratios, mixing time, and reaction conditions), to achieve the synthesis of NPs with the required properties.^120,121^ Scaling up these processes while maintaining consistent and reproducible results at large volumes can be complex and time-consuming. Integrating microfluidic systems into existing pharmaceutical manufacturing processes and infrastructures can also represent an obstacle. Adapting and aligning the technology with current production systems, quality control standards, and regulatory requirements may require significant modifications to the production setup and additional validation.^122^ Furthermore, microfluidic devices are often delicate and sensitive to variations in their operating conditions (e.g., correct functioning of the automated syringe pumps), which can lead to inconsistent performance and reliability issues. Thus, this obstacle must also be overcome, since large-scale production demands robust and reliable systems that can operate continuously without frequent maintenance or interruptions.

Various strategies have been explored to overcome the above-discussed limitations. For instance, the scaled-up synthesis of hollow gold NPs (HGNPs) using both batch and microfluidic device approaches was investigated.^123^ Scaling up of the batch synthesis to a volume of 1.2 L (10-fold increase) led to the production of nonhomogeneous HGNPs suspensions and a decreased yield production. The inefficient mixing as well as the increase of the reaction volume likely contributed to the poor quality of the HGNPs in the scaled-up batch synthesis. Conversely, scaled-up production was successfully achieved by increasing both the diameter and length of the reactor. It led to a 10-fold increase in throughput, while maintaining the production of HGNPs with the same desirable features.

**Figure 5:**
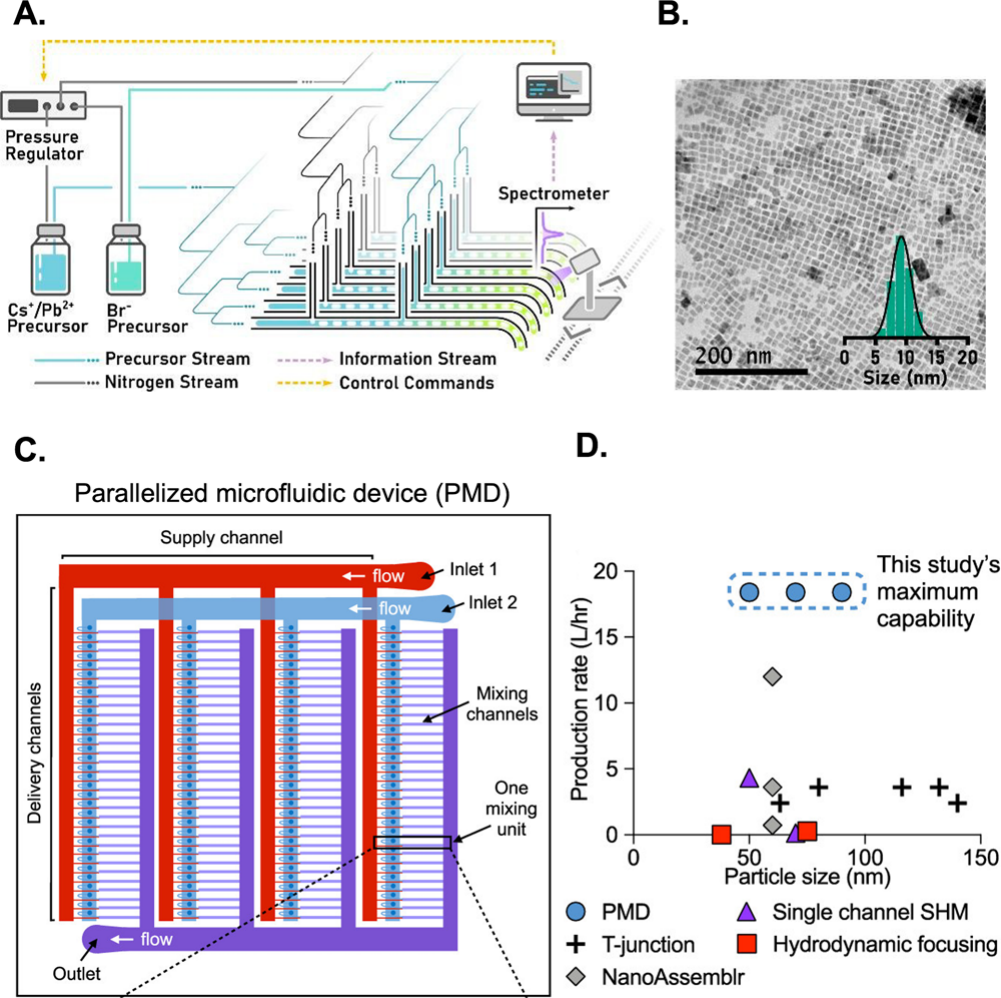
Schematic representation of a 16-channel microfluidic reactor equipped with an integrated system for photoluminescence monitoring (A - Reprinted with permission from ref (125). Copyright 2020 Royal Society of Chemistry). The resulting quantum dots obtained from this reactor exhibited a consistent average size of ∼10 nm (B - Reprinted with permission from ref (125). Copyright 2020 Royal Society of Chemistry). The parallelized microfluidic device incorporates an array of 128 mixing channels (C - Reprinted with permission from ref (126). Copyright 2021 American Chemical Society). The production rate of this parallelized microfluidic device (PMD) was compared to alternative approaches, with a focus on the total volumetric production rate and the corresponding size of lipid NPs (D - Reprinted with permission from ref (126). Copyright 2021 American Chemical Society).

A different approach for scaling up the NPs production involves the parallelization of microfluidic systems, wherein multiple devices are operated simultaneously to enhance sample throughput.^124^ Unlike enlarging channel sizes, which can impair heat and mass transfer or increasing flow rates that may lead to inadequate residence times, as previously presented, the parallelization strategy offers an appealing solution for achieving a high-throughput synthesis of NPs. This approach maintains the stability of the reactor geometry while enabling high throughput through linear scaling by increasing the number of channels. On this topic, a multiphase flow system made of a 16-channel microfluidic reactor was used for the continuous production of CsPbBr3 quantum dots.^125^ The reactor consisted of a 3D-printed manifold featuring one inlet and four outlets, enabling the uniform distribution of fluids (Figure 5A). The fluid, whether it be liquid or gas, is initially introduced into a single manifold and then evenly distributed into four downstream manifolds. This distribution process ultimately divides the flow into a total of 16 channels. The obtained quantum dots showed a consistent average size of ∼10 nm, demonstrating the uniformity of the samples (Figure 5B). Furthermore, the parallelized setup demonstrated a 10-fold increase in production yield compared to the nonparallelized configuration (1 vs 0.1 L/h).

High productivity can also be achieved in the case of lipid-based NPs. Indeed, a scalable parallelized microfluidic device featuring an array of 128 mixing channels was used to enhance the throughput of lipid NPs.^126^ This innovative device incorporated an array of staggered herringbone micromixer channels and flow resistors to operate simultaneously (Figure 5C). This approach built upon the established advantages of microfluidic lipid NPs production, including the reproducible synthesis of small-sized NPs (<100 nm) and low polydispersity. Furthermore, by implementing the parallelized microfluidic device, a production rate >100-fold compared to single microfluidic channels without sacrificing the desirable lipid NPs’ physical properties was achieved (Figure 5D).

Overall, the progress made in the development of microfluidic reactors with customized features, including channel size enlargement, parallelization, and the implementation of continuous-flow processes, offers significant promise in achieving higher production rates compared to traditional processes. These innovations hold the potential to revolutionize production methods and enhance scalability.

### 2.2 Microfluidic Devices for in Vitro Models

Traditionally, cells have been cultured in flasks, Petri dishes, or well plates, where they can grow, proliferate, and be used in different assays. However, these assays are performed in static conditions, and cells’ interaction with the surrounding environment is limited with important repercussions in cell phenotype, functionalities, and response to stimuli.^127,128^ Indeed, in the traditional setup, cell-environment interactions and physiological parameters are not easy to mimic. Consequently, more representative in vitro models are required to predict the performance of NPs in vivo. Microfluidic devices for cell culture are a valuable bridge between traditional in vitro assays and in vivo conditions. These devices provide a more representative physiological environment compared to 2D assays, enabling us to study a particular phenomenon in models that closely resemble those found in living organisms. Indeed, they offer a valuable alternative for studying, e.g., cellular behavior, drug responses in disease models, providing meaningful insights, and generate more straightforward and conclusive human-related data that contribute to reducing the number of animals used in research.^129−131^ Microfluidic-based cell cultures present numerous advantages, such as allowing a dynamic and controlled environment (e.g., chemical gradients, flow rate, shear stress, pH, CO_2_, O_2_, or temperature), the continuous inflow of nutrients by perfusion, regular waste removal, less consumption of fluids, automated liquid handling systems (e.g., pumps), and the possibility of integration with biosensors.^132^ Therefore, microfluidic systems are increasingly being used as resourceful tools and valuable alternatives to traditional approaches. Figure 5 summarizes the main advantages and disadvantages of static versus dynamic culture systems.

Microfluidic chips for cell cultures allow for precise engineering of the cellular architecture in the micron-scale range.^133^ This enables building specific designs that resemble physical and chemical microenvironments to answer specific challenges (e.g., the blood-brain barrier, vascular circuits, extravasation, or tumor permeation). Indeed, microfluidic platforms can be designed for several specific applications, such as single-cell studies,^134,135^ cell trapping,^136,137^ cell filtration,^138^ cell rolling,^139,140^ cell migration,^141^ drug screening and discovery,^142^ biomarkers detection,^143,144^ organ-on-a-chip,^145^ and body-on-a-chip.^146,147^ Moreover, microfluidic devices operating in a continuous-perfusion mode allow for enhancement and optimize the microenvironment for cell functions. Indeed, as previously mentioned, this dynamic condition allows for the efficient delivery of nutrients and oxygen to the cells while metabolic wastes are removed. Fluid flow also needs to be fine-tuned since cells can respond to physical cues and transform them into a biological response (cellular mechanotransduction).^148^

Nonetheless, adopting the microfluidic technology for in vitro assays may present a number of obstacles. One inconvenience can be related to the difficulty of use, which can make operation and deployment problematic (e.g., assembly of the microfluidic setup, chip handling, and tubing and lever taper arrangement). Furthermore, the cost of microfluidic chips frequently hinders their wide adoption. Another obstacle is the lack of established protocols among various microfluidic devices, which makes it difficult to seamlessly integrate components and transfer assays between platforms. Conversely, the development of standard interfaces and protocols would substantially aid the widespread use of microfluidic technology. Furthermore, technical issues, such as the formation of air bubbles into channels and the precise maintenance of culture media temperature and pH, may affect the high throughput and scalability of these platforms. However, significant scientific advancements have enabled companies such as MIMETAS and CN-Bio to develop innovative products that effectively tackle these challenges. These companies have dedicated their efforts to provide customized and high-throughput solutions for diverse applications, with a specific focus on improving reproducibility and scalability in microfluidic cell culture.^149−151^

**Figure 6:**
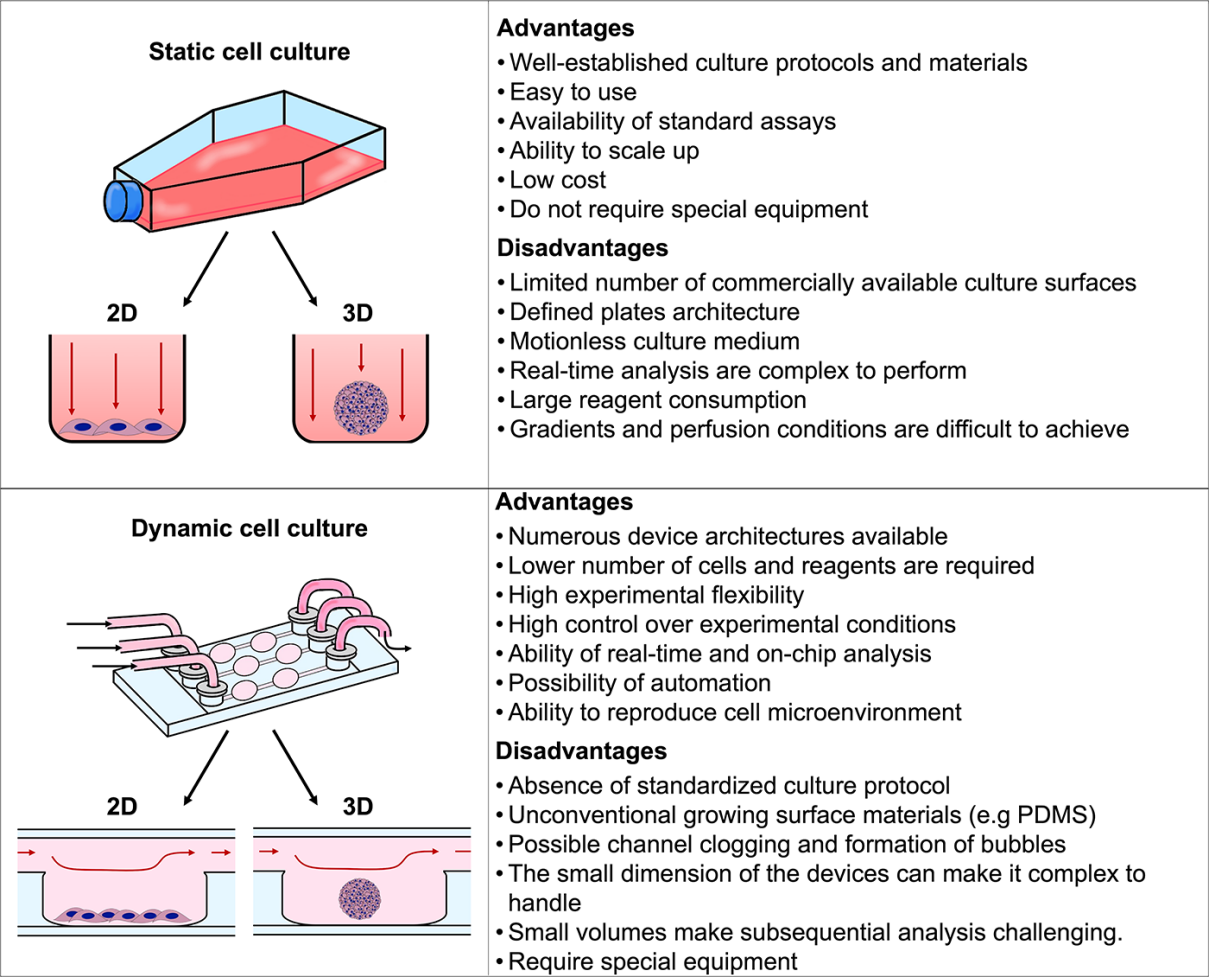
Schematic analysis of the advantages and challenges of both static and dynamic cell cultures.

In this context, organ-on-a-chip represents an advanced in vitro platform mimicking the characteristics of body tissues and organs. These devices are built using a reductionist approach since they aim to replicate the main features of the specified organ by using the appropriate microchannel architecture with specific cell types.^152−154^ Organ-on-a-chip systems offer a powerful platform to evaluate NPs’ toxicity on specific tissues. Some examples of their applications will be discussed in the following sections.

#### 2.2.1 Vascular Barrier

**Figure 7:**
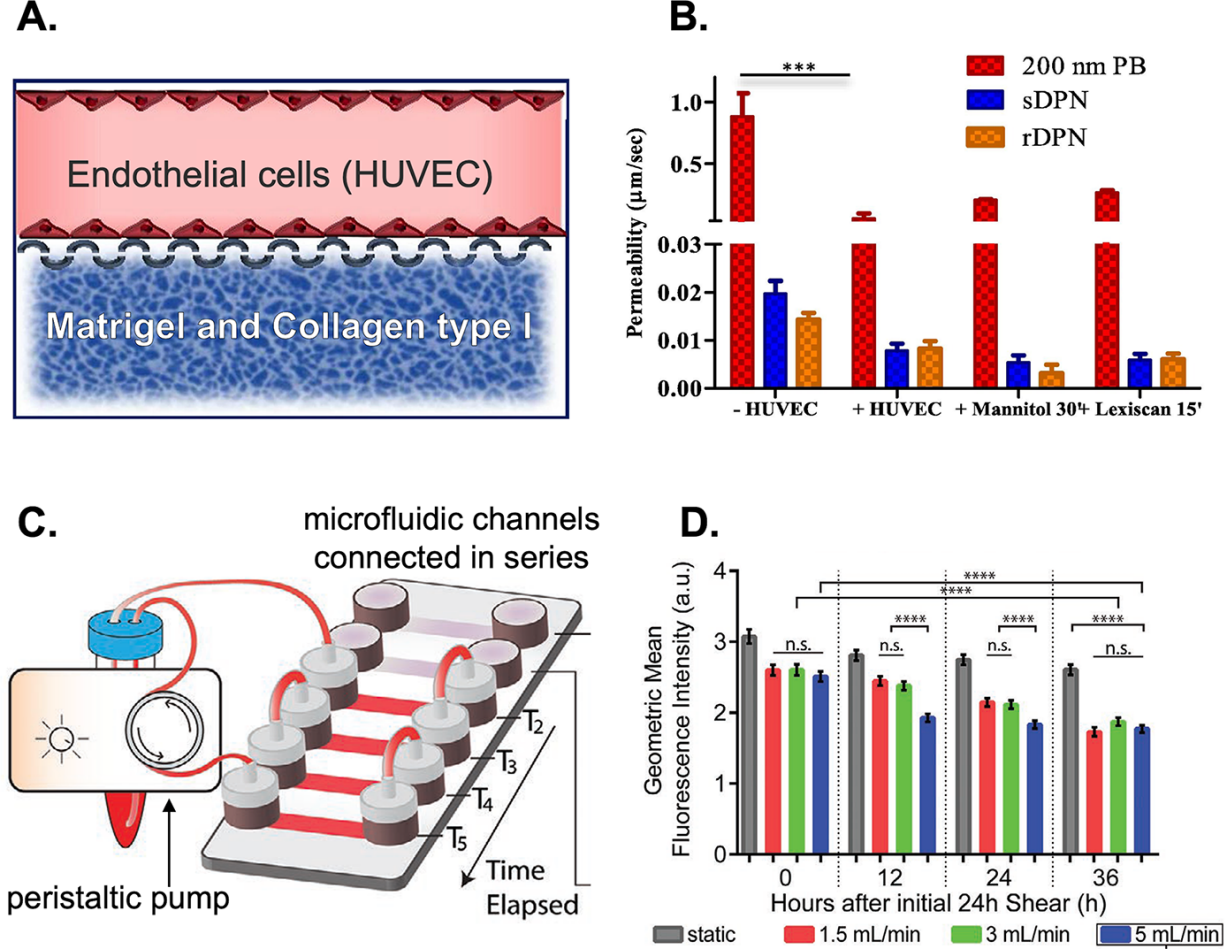
Double-channel microfluidic device showing the vascular channel, seeded with HUVEC, and the extravascular chamber filled with Matrigel and collagen type I to represent the extracellular matrix (A - Adapted with permission from ref (164). Copyright 2021 Elsevier). Vascular permeability of polymeric nanoconstructs namely 200 nm polystyrene NPs (PB), soft discoidal polymeric NPs (sDPN), and rigid discoidal polymeric NPs (rDPN) in the absence of HUVEC (−HUVEC), with HUVEC (+HUVEC), with HUVEC treated with 1 M mannitol for 30 min and with HUVEC treated with 1 μM Lexiscan for 15 min (B - Reprinted with permission from ref (164). Copyright 2021 Elsevier). Microfluidic chip incorporating a series of six interconnected channels, which are linked to a peristaltic pump and a media reservoir (C - Adapted with permission from ref (165). Copyright 2020 Wiley). The uptake of NPs by HUVECs presents changes upon shear adaptation. HUVECs exposed to high shear rates have decreased capacity to uptake untargeted NPs (D - Reprinted with permission from ref (165). Copyright 2020 Wiley).

To study the NPs’ distribution and their ability to cross different biological barriers, several microfluidic platforms mimicking specific scenarios can be employed. These models have been applied to evaluate NPs margination^155^ and extravasation,^156^ as well as the effect of shear stress.^157,158^ Indeed, the NPs’ ability to cross biological barriers is an important subject of study.^82,159^ The efficacy of the majority of intravenously administered NPs depends on their ability to cross the vascular endothelial barrier before diffusing toward their final target organ/tissue. Thus, different microfluidic models were developed to assess vascular permeability to NPs.^160−162^ As such, the impact of the protein corona in the cellular uptake and transcellular permeability of polystyrene NPs was evaluated in a microfluidic channel resembling the microvasculature environment.^163^ Fetal bovine serum was selected to incubate with NPs of 20, 40, 100, and 200 nm. The outcomes showed that the protein corona affected NPs uptake and transcytosis in a size-dependent way. Also, the selective targeting of caveolae-mediated endocytosis may not necessarily enhance transcytosis, and the cellular uptake of NPs did not fully recapitulate their transcytosis rate. Indeed, large NPs (100–200 nm) showed the highest uptake but the lowest transcellular crossing. In addition, a study used a microfluidic vasculature model to evaluate the permeability of macromolecules and polymeric NPs in physiological and pathological conditions.^164^ The dual-channel microfluidic device was engineered to include both vascular and extravascular compartments, which were connected through a micropillar membrane (Figure 7A). The upper channel was covered with a continuous layer of endothelial cells, while the lower channel was filled with a matrix. The results show how the system can be modulated by using two clinically relevant agents (mannitol and lexiscan) to regulate vascular permeability by reproducing specific physiological conditions. Moreover, they could promote the perivascular accumulation of NPs of approximately 200 nm in a dose- and time-dependent manner while having no effect on larger particles. Furthermore, the device was used to study the deformability of NPs in a vascular dynamic assay using soft and rigid discoidal polymeric NPs (Figure 7B). The results showed that soft NPs can adhere more efficiently to vascular walls than rigid formulations under pathological conditions. Additionally, microfluidic systems can be exploited to investigate the efficacy rate of functionalized carriers in a customized microenvironment that better mimics an in vivo scenario. As such, a microfluidic platform to evaluate if NPs’ functionalization increases their uptake by endothelial cells under different flow conditions was developed.^165^ For that, Au NPs of 100 nm were conjugated on the surface with Ulex Europaeus Agglutinin I (UEA-1) lectin, which binds to human endothelial cells. To investigate this phenomenon, we utilized a microfluidic platform consisting of six interconnected channels (Figure 7C). Results showed that the NPs’ uptake changed upon shear adaptation (Figure 7D). Additionally, it was observed that untargeted NPs did not undergo internalization by endothelial cells when subjected to flow conditions. Also, significant uptake was observed only under static conditions. Conversely, surface functionalization enhanced NPs’ ability to interact with cells and, thus, increased their internalization rate under dynamic conditions. A microfluidic device mimicking dysfunctional endothelium was also developed to screen the targeting efficacy of different VCAM-1-binding NPs under pathological shear stress conditions.^166^ Results showed that the smaller NPs (∼50 nm) demonstrated a higher permeability and binding efficacy.

Finally, toxicity studies have A huge importance due to the possible damaging effect of nanomaterials on cells, tissues, and organs.^167^ These studies require high-throughput screening methods as the toxic effects of a nanomaterial can be due to different factors (e.g., composition, size, shape, or surface modification). Moreover, different cells, tissues, and organs of the human body may react differently after exposure to a given nanomaterial. This results in endless combinations that an ideal toxicology screen should test. Thus, the demand for fast and robust screening platforms is rising, and organs-on-a-chip may be the answer for enhanced and more accurate toxicity screening tools. Their use in toxicity screening assays brings important advantages, such as reduction of the sample volumes, reduced costs, precise control over the flow parameters, and the possibility to customize the design or functionalization of the microchannels. In this context, microfluidic devices with different designs were proposed. For instance, a linear single-microchannel was used to assess the cytotoxicity of ∼50 nm mesoporous silica NPs.^168^ A shear-stress-dependent toxicity was observed for endothelial cells. A similar device was also employed to investigate Au NPs cytotoxicity.^169^ Results revealed that the administration of ∼7 nm Au NPs under flow conditions reduced their sedimentation and aggregation, resulting in lower cytotoxicity compared to experiments performed in static conditions in multiwells.

#### 2.2.2 Blood–Brain Barrier

Another important vascular barrier is the blood–brain barrier (BBB), which is a highly selective semipermeable vascular barrier that regulates the transport of substances between the blood and the brain. In order to recapitulate the key structure and function of the human BBB, a microphysiological platform was designed to investigate the biodistribution of NPs in this 3D environment.^170^ For this study, high-density lipoprotein mimetic NPs were synthesized using a microvortex propagation mixer with an intensity peak of ∼50 nm. Using this BBB chip, the authors demonstrated the potential use of the developed NPs as drug delivery systems due to the enhanced ability to cross the BBB via scavenger receptor-B1-mediated transcytosis. Additionally, they demonstrated the on-chip mimicry of the BBB structure and function by cellular interactions, key gene expression, low permeability, and 3D astrocytic network biodistribution. Moreover, they provided evidence that the BBB-on-a-chip enables multiple analyses, such as TEER measurement, NPs sampling, imaging, and FACS analysis, making it a useful tool for translational drug delivery research.

#### 2.2.3 Mucus Barrier

The mucus covering the epithelial tissues of several organs, such as the lungs, vagina, eyes, nose, and gastrointestinal tract, represents an important biological barrier. It is characterized by high viscosity, the NPs distribution and uptake being very different from other biological barriers. As such, the understanding of the mechanisms driving the NPs’ transport across mucus layers can be evaluated using microfluidic devices, which aid to accelerate NP optimization and development.^171−173^ Therefore, a mucus-on-chip was designed to quantify the transport of NPs across mucus.^174^ This approach enabled visualizing in real-time the penetration of ∼50 and 200 nm NPs. The results showed that NP migration across the chip was size- and surface functionalization-dependent. Indeed, PEGylation significantly enhanced the penetration of both NPs, while a pectin coating limited their passage. Additionally, this platform can be tuned to simulate specific physiological mucus environments. For instance, the treatment with a mucolytic agent decreased the mucus barrier, and thus, NPs migration was accelerated regardless of their size and surface functionalization.

#### 2.2.4 Placenta Barrier

Microfluidic models were developed to study the NPs’ permeability through the placenta.^175−178^ Indeed, the use of therapeutic agents during pregnancy is complex, as care must be focused on the mother and not compromise the fetus’ health. Consequently, it is of extreme importance to consider any possible fetal toxicity, teratogenicity, and long-term effects on newborns that maternal drug treatments can cause. In this context, an in vitro 3D placental barrier-on-a-chip microdevice was developed to resemble the maternal and fetal interface and evaluate the effect of TiO_2_ NPs exposure.^179^ It was demonstrated that 50 nm TiO_2_ NPs accumulate and transfer across the trophoblastic layer but barely cross the fetal endothelial layer. Regardless of the lack of NPs transfer to the fetal compartment, several parameters were investigated, namely the barrier integrity and permeability, cell apoptosis, production of reactive oxygen species (ROS), and adhesion of maternal macrophages. When the system was exposed to low concentrations of NPs, no significant alterations in ROS production and cell death were observed. However, placental barrier dysfunction and altered immune cell behavior were identified, suggesting potential TiO_2_ NPs-induced damage.

#### 2.2.5 Tumor-on-a-Chip

**Figure 8:**
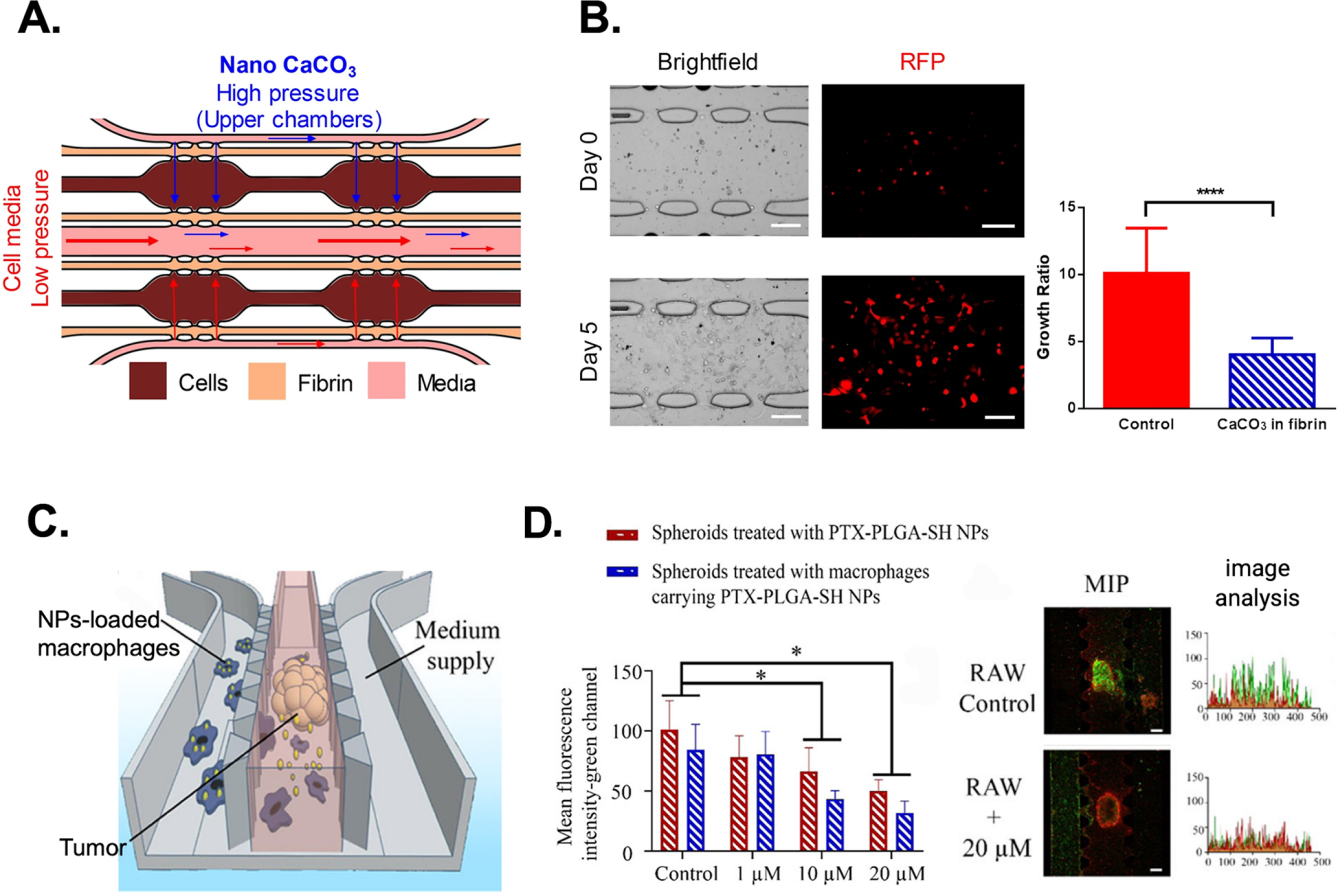
A microfluidic device consisting of three sections was used to investigate tumor migration. The brown present MDA-MB-231 cells loaded in fibrin gels, while the adjacent chambers contain plain fibrin for measuring cellular migration. Inside the pink channel, culture media was flowing to nourish the tissue. The upper chambers will receive media containing CaCO_3_ nanoparticles, whereas the lower chambers will receive plain media (A - Reprinted with permission under a Creative Commons CC BY License from ref (190). Copyright 2021 Springer Nature). The treatment with nanoCaCO_3_ caused inhibition of breast cancer MDA-MB-231 cell line growth and migration (B - Reprinted with permission under a Creative Commons CC BY License from ref (190). Copyright 2021 Springer Nature). The microfluidic device integrates three microchannels separated by two lines of trapezoidal PDMS pillars. This setting enables the independent loading of hydrogel into each channel, facilitating the cultivation of tumor spheroids and macrophages in separate compartments while allowing substance exchange and intercellular crosstalk (C - Reprinted with permission from ref (192). Copyright 2020 American Chemical Society). Confocal images showing the cell viability of tumor spheroids and corresponding image analysis. The spheroids treated with PTX-NPs-macrophages exhibited higher mortality rates compared to the treatment with PTX-NPs alone (D - Reprinted with permission from ref (192). Copyright 2020 American Chemical Society).

In cancer research, 3D culture models are more representative of the tumor microenvironment than 2D cultures, tumor spheroids being the most popular approach due to their reproducibility and simplicity of production.^180,181^ The combination of tumor spheroids with microfluidic systems advanced the concept of in vitro models. Indeed, microfluidic platforms enable the control and modulation of the culture microenvironment in terms of chemical gradients, oxygen, pH, temperature, fluid flow, and pressure. Thus, they allow for better replication of several parameters that influence in vivo NP delivery. Microfluidic devices already demonstrated their potential in the study of angiogenesis,^182^ metastasis,^183^ isolation,^184^ drug screening,^185^ and NP penetration^186^ and uptake.^187^ The delivery of NPs to the tumor bed is a multistep process that requires overcoming several challenges, such as vessel extravasation, target specificity, tumoral heterogeneity, and cellular internalization for the delivery of the therapeutic agents.^188^ Indeed, the efficiency of transport, tissue or organ targeting, and accumulation of NPs can be investigated using tumor-on-a-chip devices.^189^ For instance, the effect of calcium carbonate NPs on tumor survival and migration was studied using a microfluidic device.^190^ This chip was designed with a bifurcated geometry that allowed us to closely compare two cell environments and to control interstitial flow rates (Figure 8A). Additionally, the fluid flow rates and their directions were determined by differences in pressures along the channels. NPs in this model demonstrated their therapeutic effect by buffering the extracellular pH, which caused inhibition of breast cancer MDA-MB-231 cell line growth and migration (Figure 8B).

Another important factor is the characterization of the NPs diffusivity and permeability in the tumor microenvironment. The traditional use of 2D and 3D in vitro models under a static environment provides only limited information by failing to realistically replicate the interaction of the NPs with the surrounding elements. Consequently, these studies provide insufficient predictive power of the behavior in vivo. Conversely, microfluidic systems offer opportunities for NPs’ evaluation in physiological conditions by mimicking the microenvironment of different tumors. Thus, microfluidic tumor models are well suited for modeling and studying specific events. For instance, to assess the penetration of NPs into the cancer cell mass a microfluidic device for coculture of 4T1 breast cancer cells and EA.hy926 endothelial cells was employed.^191^ These cell types, selected to resemble the tumor microenvironment, were exposed to nanocrystals (∼310 nm in length) under physiological shear stress. The results demonstrated the impact of the endothelial cell barrier on NPs penetration. In fact, while NPs readily diffused into the center of the tumor in the absence of the endothelial layer, minimal penetration was seen in its presence. A similar conclusion was obtained in another study.^160^ The data obtained also confirmed the permeable nature of the tumor vascular system in which more NPs were absorbed by cells localized near the junctions of the endothelial gap than by cells far from the junctions. Additionally, a tumor-microenvironment-on-a-chip composed of tumor spheroids embedded in a collagen gel was developed in order to study the infiltration of macrophages carrying NPs.^192^ Polymeric NPs loaded with paclitaxel were internalized in macrophages, and then, the cells were introduced in the microfluidic side channel to evaluate their migration toward the tumor spheroids (Figure 8C). They demonstrated that macrophages improved the therapeutic efficacy of the incorporated NPs by facilitating drug delivery into the inner tumor regions (Figure 8D).

Finally, NPs can also be designed to target specific tumors based on their organ of origin. For instance, a microfluidic chip was designed to recapitulate the tumor microenvironment and assess the ability of NPs to specifically target cancer cells.^193^ Folic acid-cholesterol-chitosan NPs of 100 nm were tested on human lung adenocarcinoma (A549) and cervical cancer cells (Hela). The fluorescence images showed the targeting ability of the studied NPs toward HeLa cells compared to A594 cells. The robustness of the designed chip for in vitro screening was further proven by in vivo testing, indicating that the developed NPs showed targeting for folate receptor-positive tumors.

The use of microfluidics-based tumor models in NP research provides valuable insights due to their ability to closely mimic pathophysiological environments. Consequently, critical aspects of NPs behavior, such as tumor targeting and permeability and accumulation in the tumor microenvironment, are addressed in conditions that allow for enhancing the understanding of in vivo processes. Thus, microfluidics contributes to the development of more realistic in vitro models, leading to a more accurate screening of NP-based cancer therapeutics.

#### 2.2.6 Lung

At present, the evaluation of pulmonary toxicity caused by NPs relies heavily on cell culture and animal models. The in vitro models enable quantitative evaluation of nanomaterial toxicity and the generation of mechanistic insights specific to different cell types. Thus, the outcomes obtained from these in vitro assays do not fully recapitulate what is observed in vivo due to the absence of cellular architecture, such as the alveolar-capillary barrier and microenvironmental cues. To address these limitations, various microfluidic models were developed to tackle specific research questions. For instance, the alveolar-capillary barrier was reproduced on a microfluidic device in order to assess the nanotoxicity of TiO_2_ NPs and ZnO NPs.^194^ The device consisted of three parallel channels for the coculture of human vascular endothelial cells and human alveolar epithelial cells separated by a Matrigel membrane. Results showed that TiO_2_ NPs did not induce significant toxicity, while the same treatment performed with ZnO NPs led to ∼50% apoptosis in epithelial cells and ∼5% in endothelial cells. Additionally, a multifunctional microdevice to effectively replicate the essential structural, functional, and mechanical properties of the human alveolar-capillary interface was developed.^195^ In this study, a two-channel microfluidic device with a porous membrane coated with collagen was utilized. This membrane acted as a barrier, separating the alveolar epithelial cells in the top channel (in contact with air) from the microvascular endothelial cells in the bottom channel (in contact with perfused cell culture medium). Once the cells reached confluence, air was introduced into the epithelial compartment, creating an air–liquid interface that closely resembled the lining of the alveolar air space in the human lung. Finally, the impact of airborne exposure to toxic nanomaterials was assessed by introducing silica NPs into the system. The results demonstrated that artificial respiration within the microdevice induced greater transport of NPs from the epithelial to the endothelial channel. This led to a greater uptake of NPs by the endothelial cells compared to that of the tissue layers cultivated in submerged liquid culture conditions. This increased uptake of NPs was also associated with the enhanced expression of intercellular adhesion molecule-1 (ICAM-1) and the production of reactive oxygen species (ROS). These findings suggested that the inspiration of NPs exacerbated the development of acute lung inflammation. By employing this advanced microdevice, researchers were able to gain valuable insights into the effects of NPs on lung tissue under conditions resembling physiological respiration. Ultimately, lung-on-a-chip devices hold promise for further understanding the mechanisms behind NP-induced lung inflammation and can contribute to the development of safe nanomaterials and improved respiratory health.

#### 2.2.7 Heart

Unfortunately, as demonstrated by the currently limited literature, advances in heart-on-a-chip for NP screening are not as pronounced as those observed for other organs. However, a study has delved into investigating the adverse effects of copper oxide (CuO) and silica (SiO_2_) NPs associated with air pollution, utilizing a heart-on-a-chip model.^196^ Endothelial cells and iPSC-derived cardiomyocytes were seeded onto the microfluidic bioscaffold, which featured a distinctive pattern of 15 μm microholes on the vessel wall. This design enabled the transport of macromolecules and NPs into the parenchymal tissue and facilitated intercellular communication. In this model, CuO NPs had the ability to disrupt the endothelial barrier and translocate into cardiac tissue, leading to alterations in its function. Furthermore, CuO NPs generated significant levels of reactive oxygen species (ROS), contributing to cardiac injury. Conversely, SiO_2_ NPs did not generate notable levels of ROS and did not significantly affect endothelial cell junctions. However, SiO_2_ NPs were able to indirectly modulate cardiac function by triggering the secretion of pro-inflammatory cytokines. Ultimately, heart-on-a-chip holds significant potential for both pharmacological and disease modeling applications, especially when integrated registration systems for contraction and action potential are utilized. Consequently, it is crucial to conduct further investigations into these platforms, specifically regarding assessing the toxicity and therapeutic efficacy of NPs.

#### 2.2.8 Spleen

A spleen-on-a-chip was created to cleanse the blood of sepsis patients by employing nanobeads coated with opsonins.^197^ Incorporating innovative architectural elements reminiscent of the spleen, the microfluidic device comprised a high-flow vascular arterial channel, which was perfused with contaminated whole blood, alongside a parallel venous sinusoid channel with low or intermittent flow. These two channels were interconnected through openings, resembling separation of the arterial red-pulp cord and venous systems by sinusoid slits. By adding to the contaminated blood magnetic nanobeads coated with an engineered human opsonin-mannose-binding lectin, the magnetic separation process effectively eliminated pathogens. Consequently, the venous sinusoid channel facilitated the removal of the pathogens, while the arterial channel retained purified blood.

#### 2.2.9 Kidney

So far, there is a lack of literature documenting the utilization of kidney-on-a-chip (K-on-a-chip) for NPs screening. Nonetheless, there is a study that investigated the use of fluorescently labeled NPs for kidney injury imaging.^198^ By introduction of fluorescent polystyrene NPs coated with anti-γ-glutamyl transpeptidase (GGT) antibodies into the apical channel, drug-induced nephrotoxicity was effectively monitored. Indeed, the NPs exhibited enhanced fluorescence in the outflow as they aggregated upon capturing this protein, which is released in response to proximal tubular cell injury. Notably, a smartphone-based fluorescence microscope was integrated into the chip, enabling convenient and portable monitoring of the kidney-on-a-chip. Consequently, this approach provides a solution to the challenges associated with rapid, continuous, and noninvasive assessment of biological responses during experiments.

#### 2.2.10 Liver

In-vivo studies on distribution of administered NPs, showed that the liver acts as a filter and enhances their clearance. Consequently, NPs can accumulate in this organ, causing liver damage. On this matter, a 3D hepatocyte chip was developed for hepatotoxicity testing of NPs.^199^ The 3D hepatocyte chip recapitulated the key physiological responses related to hepatotoxicity. The results were compared with the NPs exposure in static conditions, using multiwell plates. The hepatocytes subjected to cumulative exposure under static conditions exhibited more severe damage, highlighting the significance of testing NPs’ inaccurate data. A significant advancement in this field was also achieved by combining a liver-on-a-chip with an intestine-on-a-chip.^200^ This system was constructed by incorporating a coculture of enterocytes (Caco-2) and mucin-producing cells (TH29-MTX) to represent the human intestinal epithelium, along with HepG2/C3A cells to represent the liver, in a single microfluidic device. Despite the intestine tissue acting as a substantial barrier to the NPs, the findings revealed that 50 nm carboxylated polystyrene NPs caused cellular damage in the liver. Interestingly, the presence of the intestine tissue upstream of the liver introduced additional factors that exacerbated the injury such as changes in the NPs’ properties as they crossed the intestinal tissue. Indeed, the NPs collected from the basolateral side after 24 h exposure exhibited a decrease in the magnitude of the zeta-potential (∼ −12 mV) in contrast to the NPs incubated in a cell culture medium for the same time (∼ −18 mV). Additionally, a variation in NPs size distribution was also observed (∼97 nm in the apical side versus ∼55 nm in the basolateral side). This indicates NPs’ size alteration while interacting with the cellular layer (sizes of NPs stored in culture medium and water are ∼97 and ∼40 nm, respectively). Thus, the use of this device enhanced the sensitivity in evaluating NP-induced injury, providing more realistic data compared with experiments conducted solely on a single tissue.

### 2.3 Microfluidic for Organism-on-a-Chip

Fish, flies, and worms have been widely used due to their specific characteristics, such as small size, optical transparency, and relatively short life span, that make them suitable for research studies. Moreover, they are well-characterized in terms of anatomical structure, genome, and manipulation. However, the traditional strategies based on macroscale tools are not adequate for the handling of these organisms and turn out to be demanding and time-consuming, which translates into reduced throughput and limited discovery speed. To overcome these limitations, many microfluidic devices have been developed that allow for organism immobilization and experimentation.

Despite being in its infancy, the use of microfluidics for NPs testing in animal models will enable a detailed study of multiple cellular and subcellular phenomena in living organisms over different developmental stages. Indeed, microfluidic technologies can be applied for the phenotyping and screening of small model organisms such as nematodes, fruit flies, and zebrafish. These small organisms have mainly been used to acquire knowledge about embryonic development. For example, the zebrafish (*Danio rerio*) has been applied to study the genetic effects of human diseases and drug screening.^201−203^ The fruit fly (*Drosophila melanogaster*) was extensively used to study genetic mutations, heredity, as well as biological processes, including embryonic development, learning, behavior, and aging.^204−206^ Finally, the nematode *Caenorhabditis elegans* has been used as a model for research in molecular biology, medicine, pharmacology, and toxicology.^207−210^

**Figure 9:**
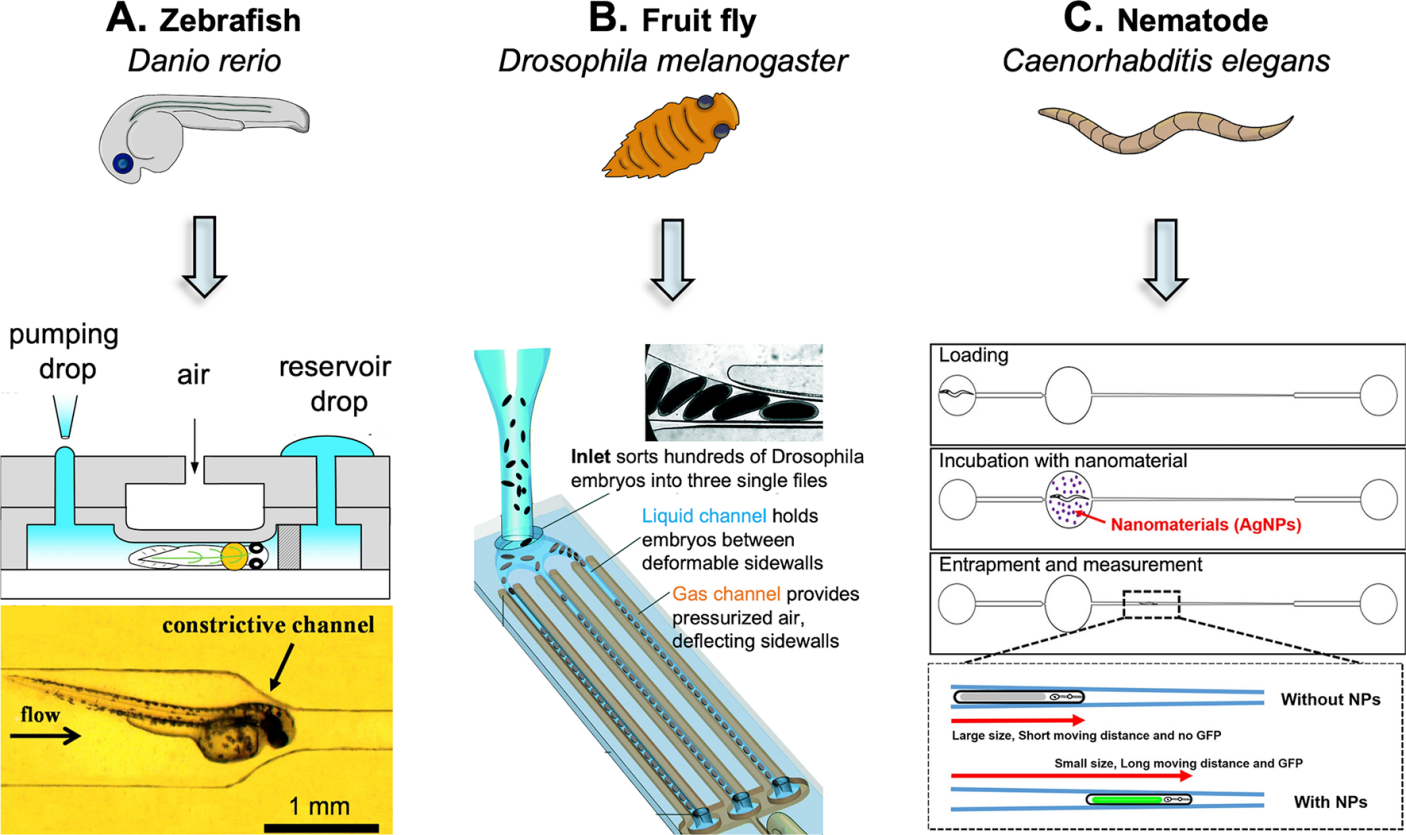
Illustration of microfluidic technology application for small animal testing. Microfluidic devices have been developed for the accurate handling of zebrafish (A - Reprinted with permission under a Creative Commons CC BY 4.0 License from ref (225). Copyright 2015 MPDI), fruit fly (B - Reprinted with permission from ref (222). Copyright 2019 Royal Society of Chemistry), and nematode (C - Reprinted with permission under a Creative Commons CC BY License from ref (224). Copyright 2017 Springer Nature).

The zebrafish *(Danio rerio*) is a freshwater fish extensively used as a vertebrate model organism in scientific research. The regenerative ability of this fish stands out, being widely investigated. Moreover, as its genome is fully sequenced, transgenic strains can be produced to investigate different diseases. In particular, the use of zebrafish aids to study and unlock several biological processes behind muscular dystrophy. Microfluidics allows us to efficiently entrap this organism, which translates into high-resolution imaging within a specific temporal–spatial condition (Figure 9A). On this matter, microfluidic platforms with different devices were designed for the behavioral screening of zebrafish larvae.^211^ This study demonstrated that microfluidic technology can be used to reduce the time of behavioral screening and facilitate the screening of larger sample sizes with an electrical stimulation method. Indeed, the most performing platform designed enabled the loading in parallel of four larvae loading, their partial immobilization (to allow tail movements), exposure to an electric stimulus, and the possibility to quantify the tail movement induced by the electric input.

In addition to the geometry of the channel, alternative entrapping techniques are represented by the appliance of gravity force,^212,213^ suction force,^214,215^ and droplet encapsulation.^216^ Indeed, in order to improve the embryo handling and manipulation of zebrafish, a two-plate droplet-based “digital” microfluidic technology for on-chip transporting of zebrafish embryos was developed.^217,218^ Additionally, on-chip high-quality imaging was developed to achieve fine-tuning of temperature, light, and oxygenated water levels, remote transport and orientation through light patterning, and dynamic culturing of zebrafish.^219^ This noninvasive automated system eliminated manual handling, enabling more accurate imaging measurements.

The fruit fly (*Drosophila melanogaster*) is a multicellular model organism frequently used as model in developmental biology, being an excellent genetic tool with low cost and rapid generation time. The manipulation of *D. melanogaster*, including its delivery, orientation, injection, and immobilization, plays a decisive role in all biological assays. In this regard, microfluidics containing this organism was, for instance, employed to investigate the cardiac toxicity of heavy metals. Indeed, a microfluidic device was developed to expose the larvae’s hemolymph to controlled injection of zinc or cadmium and evaluate the impact on the heart rate and arrhythmicity.^220^ This study demonstrated that the developed platform can be employed to investigate the acute cardiac toxicity of heavy metals by accurate microinjection, followed by heart monitoring. Moreover, the device enabled overcoming several technical challenges, including the delicate handling of the small larva, its precise orientation, and immobilization for microinjection and heart monitoring without the use of anesthetics or glue.^221^ A further improvement in this area was provided by the development of a high-throughput device for automatic alignment, immobilization, compression, real-time imaging, and recovery of *Drosophila* live embryos (Figure 9B).^222^ Indeed, the developed platform allowed us to precisely handle hundreds of embryos and to map and quantify the responses to compression and twist during early *Drosophila* development.

Finally, *Caenorhabditis elegans* is a nonparasitic nematode characterized by a life cycle of a few days, which makes it also less complex than mammals. They easily grow, and their whole genome sequencing demonstrated a high degree of homology with human genes. For these reasons, *C. elegans* has been widely used as a model organism in biology. Microfluidic devices can also be efficiently employed for handling and immobilization of *C. elegans*, giving the advantage of abolishing the use of glues and anesthetics that are required for worm immobilization in common approaches. For instance, a microfluidic device was designed to study *C. elegans’* chemotaxis behavior for cancer detection.^223^ The device presented a two-port structure with pillars between them to increase the migration speed of *C. elegans*. Then, the chemotaxis of *C. elegans* mutants to urine from healthy and cancer patients was investigated using the device. Results showed that chemotaxis was observed in the presence of cancer patients’ urine, demonstrating that the developed device could be used for cancer tests. To date, Kim et al.^224^ were pioneers in utilizing microfluidic devices to immobilize this worm in the scope of testing nanomaterial toxicity (Figure 9C). A microfluidic chip for *C. elegans* handling that enabled us to display the changes in body growth and gene expression in response to the silver NPs was developed. It was observed that NPs exposure led to the expression of the transgenic marker DNA, mtl-2:gfp as well as a reduction in the *C. elegans* body length and width. These changes enabled the worm to travel a longer distance than the untreated control group. Additionally, the results obtained were compared with the conventional multiwell plates assay to highlight the sensitivity and selectivity of the animal-on-a-chip.

Microfluidic systems for the above-discussed organism analysis and experimentation present tremendous potential for biological breakthroughs in many fields. Indeed, this integrated approach can catalyze fundamental insights into, e.g., pathophysiological processes and drug pharmacokinetics and pharmacodynamics that will lead to huge advances in the pharmaceutical and medical fields. Undoubtedly, they have emerged as a valuable tool, surpassing traditional approaches and demonstrating increased sensitivity and selectivity in the investigation of model organisms.

## 3 Conclusions and Future Perspectives

The microfluidic field is in constant progress due to the advancement of technologies and innovative strategies. The reduced size of microfluidic devices allows for the fine manipulation of fluids at the micro- and nanolevels and enables high mass exchange and high throughput. The application of this technology for NP synthesis has as the main advantage the precise control of the physicochemical properties of the resulting NPs by the accurate tuning of flow rates, mixing times, and ratios. Chips with several geometries are commercially available, but there is also the possibility to customize them to match different needs. Compared to the conventional bulk approaches, the use of these devices for NP production offers much higher performance in terms of time consumption, quality of the samples (controlled size and size distribution), and reagents consumption. In the future, with the progress of miniaturization, more efficient devices can be developed to allow for better control over the mix, as well as improved mechanisms to avoid the clogging issues that nowadays represent one of the main drawbacks of this technique. The use of microfluidic devices for in vitro NPs testing is surely appealing and hopefully will grow over the next years in order to fill the existing gap between in vitro and animal/human experimentation. Indeed, the traditional methods for cell culturing and experimentation lack analogy with accurate physiological and biological interactions that occur in vivo. Microfluidic technology allows for the precise recreation of specific in vitro microenvironments by modeling channel geometry and exerting precise control over various parameters (e.g., temperature, pH, and gradients). These dynamic models offer a superior resemblance to human physiology compared to static culture models. Consequently, microfluidics has emerged as a compelling platform for NP screening. However, current scientific efforts have primarily focused on employing organ-on-a-chip platforms to assess NP toxicity and cell interactions (e.g., internalization and crossing mechanisms). As a future perspective, we envision advancements in organ-on-a-chip platforms that will propel the field forward. This includes the development of disease models and multiorgan-on-a-chip systems, which will significantly expand the scope of applications for assessing the distribution, safety, and therapeutic efficacy of NPs. By incorporating disease-specific models and simulating interactions between multiple organs on a single chip, researchers can create more physiologically relevant environments for studying NPs’ behavior and optimize their drug delivery capabilities. These advancements hold great potential for advancing the field and accelerating the translation of NPs from the laboratory to clinical care.

A further level of complexity can be obtained using organism-on-a-chip methods. Indeed, microfluidic devices demonstrated their ability to be used as a tool for the handling, immobilization, injection, stimulation, and imaging of small organisms, such as *D. rerio*, *C. elegans*, and *D. melanogaster*. However, so far, only one study in the literature has reported the use of microfluidic chips for organism immobilization under the scope of NP testing. As discussed in the review, the organism-on-a-chip method allows improved performance compared to conventional approaches. From a future perspective, we envisage that these microfluidic platforms will have huge potential in the nanomedicine field, giving the advantages that microfluidics devices provide in NP testing. Indeed, their application has great potential to fill the gap between in vitro and in vivo testing and humans, and the microfluidic technology would allow reduction and refinement of the use of laboratory animals and still provide reliable results that can aid in accelerating the NPs’ translation into the clinic. Hence, the consistent progress and continuous innovation in microfluidics over the years highlight its potential as a cutting-edge technology for advancing NP synthesis and enabling robust in vitro and in vivo screening. Indeed, the integration of microfluidics and nanomedicine holds great promise for revolutionizing the drug delivery field. Indeed, it can expedite the discovery of safe and effective NP-based therapies.

## Abbreviations

CCR2: CC chemokine receptor 2
CCL2: CC chemokine ligand 2
CCR5: CC chemokine receptor 5
TLC: thin layer chromatography

## Vocabulary

**Surface-to-volume ratio (S/V)**: the ratio between the surface area and the volume of an engineered material
**batch-to-batch reproducibility**: the ability of a technique to produce different lots with the same properties
**hydrodynamic flow-focusing**: a technique used to precisely control the flow of fluids by balancing the forces of different fluid streams. It occurs when fluids with different velocities are injected side by side
**high throughput**: ability to process a large number of samples or perform experiments at a high rate
**shear stress**: mechanical force generated by the friction between a liquid and the apical cell membrane
**phenotyping**: process of studying and characterizing the physical, chemical, physiological and behavioral traits of an organism.
